# Attaining High Functional Performance in Biodegradable Mg-Alloys: An Overview of Challenges and Prospects for the Mg-Zn-Ca System

**DOI:** 10.3390/ma16031324

**Published:** 2023-02-03

**Authors:** Alexei Vinogradov, Evgeniy Merson, Pavel Myagkikh, Mikhail Linderov, Alexandr Brilevsky, Dmitry Merson

**Affiliations:** 1 Department of Mechanical and Industrial Engineering, Norwegian University of Science and Technology, 4791 Trondheim, Norway; 2 Magnesium Research Center, Kumamoto University, Kumamoto 860-8555, Japan; 3 Institute of Advanced Technologies, Togliatti State University, 445020 Togliatti, Russia

**Keywords:** biodegradable magnesium alloys, implants, mechanical properties, corrosion, stress corrosion cracking, corrosion fatigue

## Abstract

This article presents a concise overview of modern achievements and existing knowledge gaps in the area of biodegradable magnesium alloys. Hundreds of Mg-based alloys have been proposed as candidates for temporary implants, and this number tends to increase day by day. Therefore, while reviewing common aspects of research in this field, we confine ourselves primarily to the popular Mg-Zn-Ca system, taken as a representative example. Over the last decades, research activities in this area have grown enormously and have produced many exciting results. Aiming at highlighting the areas where research efforts are still scarce, we review the state-of-the-art processing techniques and summarize the functional properties attained via a wide variety of processing routes devised towards achieving a desired properties profile, including the mechanical response in terms of strength, ductility, and fatigue resistance paired with biocompatibility and bio-corrosion resistance or controlled degradability. We pay keen attention to a summary of corrosion properties and mechano-chemical interactions between an aggressive environment and loaded Mg-based structures, resulting in stress corrosion cracking and premature corrosion fatigue failures. The polemic issues and challenges practitioners face in their laboratory research are identified and discussed.

## 1. Motivation

Magnesium and magnesium alloys have attracted growing attention over recent decades as potent candidates for a wide range of biomedical applications due to their outstanding combination of mechanical properties, bio-compatibility, and, most importantly, the ability to resorb in a human body [1,2,3,4,5,6,7,8]. Magnesium can dissolve in a biologically active medium without significant adverse effects; this is to say that biodegradability is a distinct attribute of magnesium alloys, which, as opposed to conventional 316L stainless steel, CoCr alloys, or Ti-based alloys used in contemporary orthopedic surgery, makes Mg-based alloys uniquely suitable for temporary deployment in a patient’s body without a need for removal after the healing process is over. Although biodegradability is, apparently, a prime advantage of Mg and its alloys, the implants made of these materials strongly benefit from the distinctive mechanical properties, including the low elastic moduli, low density, and high specific strength. Some examples of these properties of Mg-based alloys are shown in Table 1 and are compared to those in other biomedical/biocompatible materials. We should note here that the values presented in Table 1 are for navigation only, as they vary significantly depending on the material’s processing-controlled microstructure.

The Young’s modulus of Mg and its alloys (of 43–45 GPa) is reasonably close to that of cortical bone (15–30 GPa). At least the still-existing mismatch is much smaller than that for other materials such as iron, 316L steel, CoCr, or Ti and its alloys (note that recently developed β-Ti alloys also possess an excellent combination of low elastic moduli with high strength). The mismatch of elastic moduli between the bone and the implant causes stress shielding of the bone due to a disproportional load share by the bone and the implanted structure. This bio-mechanical incompatibility can, in turn, result in early implant loosening, substantial impairment to the healing process, bone atrophy, and chronic inflammation [22,23]. Being a natural element of the human body, pure magnesium is perfectly biocompatible. However, its strength (both monotonic and cyclic) and ductility are far below the numbers required to meet the demands for implant applications. Judicious alloying improves the mechanical performance of Mg a lot, making magnesium alloys comparable (or even superior) to Fe, 316LVM (medical grade) austenitic stainless steel or commercial purity (CP) Ti. Table 1 indisputably demonstrates that some Mg-alloys can be processed to extraordinarily high specific strengths in excess of 300 MPa/cm^3^/g, which is record-breaking for structural metallic materials. Leaving aside the “exotic” systems such as Mg-1Zn-2Y or heavily alloyed Mg-8Gd-3Y-0.4Zr, the popular Mg-Zn-Ca alloy also exhibits a specific strength comparable to that of Ti-alloys. A higher strength (specific strength) permits the design of implanting devices of smaller dimensions and lighter weights without compromising their load-bearing capacity (one can note that the resistance to cyclic loads may even benefit from smaller dimensions due to the well-known size effect on fatigue [24]).

Even a superficial analysis of the bibliometric data shows that hundreds of articles, including dozens of reviews, are published annually about bioresorbable magnesium alloys, and the number of publications increases drastically year by year. The whole variety of publications can be roughly and conventionally divided into four primary domains: (i) development of alloying systems and alloys with enhanced physical and mechanical properties and low cytotoxicity; (ii) thermo-mechanical processing methods enabling the utmost mechanical properties and the minimum corrosion (resorption) rate; (iii) methods for protecting the surface of implants (mainly through the use of various coatings) to minimize the rate of resorption and improve the process of bone tissue regeneration at the early stage of healing; (iv) biomedical research exploring, in depth, the interaction between the implant and living tissues (including in vitro, in vivo, and clinical examinations, etc.).

Despite the apparent advantages of Mg-based alloys for overall biomedical engineering, the widespread use of these materials in surgeon practice has been limited. The reason for this lies in the high susceptibility of Mg-alloys to corrosion and, to a lesser extent, in their inferior mechanical properties compared to those of other structural metals used as permanent implants. While the mechanical properties can be relatively easily tailored to the desired levels through the power and variety of contemporary metal processing techniques (c.f., Table 1), as will be discussed below, the focus of many studies shifted towards the design of the alloys permitting the fine-tuning of the rate of bio-degradation (or bio-corrosion) from a biological perspective [25,26]. Many Mg-alloys dissolve too quickly in a biological environment, thus causing multiple issues such as excess hydrogen evolution and the inability to keep the integrity of the bone-implant structure for the desired period. As a simple solution, many researchers propose using industrial corrosion-resistant aluminum-containing alloys of the AZ system or alloys with a high content of rare earth metals (REM) of the WE type as the base alloys. The alloy WE43 has been approved by the U.S.A. Food and Drug Administration and served as the main component for MAGNEZIX^®^ bioresorbable fixing screws by Syntellix AG (Hannover, Germany) and Lekton Magic (Biotronik, Bulach, Switzerland) coronary stents. However, concerns have been raised since the 1970s that aluminum could be a possible suspect in Alzheimer’s disease [27,28], although this is highly controversial. This long-standing suspicion has been recently challenged, since no convincing relationship between the amount of aluminum in the body and the development of Alzheimer’s disease has been established [29,30]. The etiology of Alzheimer’s disease has not been fully understood, and more research is necessary to recognize the potential of Al to cause neurotoxicity. It therefore seems prudent to avoid aluminum exposure, when practical.

The same, in principle, applies to rare elements [31]: the clinical relevance of the effect of rare earth elements dissolution on the human body is not completely clear, although extensive research activity in the field has proven many Mg-RE systems to be promising for temporary implant applications [32]. Therefore, substantial efforts have been invested in systems containing only biocompatible elements. Of the many proposed solutions, the low-alloy Mg-Zn-Ca system has gained the most significant attention by far. Leaving the early publication aside for a while, one can notice that, in 2022 alone, more than 30 articles were published about this system, including a recent review by Istrate et al. [33]. In this multifaceted research field, several works have been devoted to the study of the effect of alloying and production methods, including the fabrication of Mg-based composites based on the Mg-Zn-Ca (ZX) system [34,35,36,37,38,39,40,41]. The influence of heat treatment, metal-forming techniques relying on strain hardening, and various schemes exploiting severe plastic deformation for grain refinement and texture manipulation are described in refs. [42,43,44,45,46,47,48,49]. The relationship between the mechanical and corrosion properties and the anisotropy and texture of magnesium alloys are the focus of dedicated publications [50,51,52]. The significance of coatings for improved corrosion properties is discussed on many occasions; see [40,53,54,55,56] for examples. The in vivo response of biodegradable magnesium alloys represents a vast area of rapidly growing and promising research that cuts across many disparate fields involving materials design and engineering, biological expertise, and in-depth clinical evaluation [5]. Chaya et al. [57] have demonstrated that the degradation of the implanted Mg part does not inhibit fracture healing and enhances bone formation, supporting the potential use of Mg-based components for fracture fixation devices. Some examples of pre-clinical in vivo studies using ZX systems are given in refs. [58,59,60], suggesting a bright future for biodegradable Mg-based orthopedic implants.

One should recall that about 99% of magnesium in the human body resides in bones, muscles, and other soft tissues. The remaining 1% of magnesium in the human body is found in the blood serum or red blood cells. The standard concentration of magnesium in the blood serum of a healthy person is 0.73–1.06 mmol/L [61]. The recommended daily intake of Mg is 280–360 mg for adult women and 350–420 mg for adult men [62], which is remarkably higher than that, for example, for Fe (8–18 mg) or Zn (8–11 mg). Calcium, like magnesium, is an essential component of bones. Normal serum calcium concentrations are almost the same as those for magnesium and are 0.919–0.993 mmol/L [63]. Unlike magnesium and calcium, the total amount of zinc in the human body is relatively small, 2–3 g, with bones having a particularly high content [64]. Zinc is the second-most (after iron) abundant trace element in the body [65]; it is essential for healthy skin and the immune system. However, excessive amounts of zinc can be cytotoxic. It is therefore indispensable to keep the alloying as low as possible while managing an acceptable level of mechanical properties. Estimates show that the constituting elements of Mg-Zn-Ca alloys, when entering the human body during implant resorption, are harmless, as they cannot significantly change the natural content of body fluids and tissues; moreover, they may exert a positive effect on the treatment process [33].

Thus, there is a significant amount of literature on the medical implant applications of biodegradable magnesium alloys, which we are not going to review (re-review) here. Our goal is much narrower—a discourse on the achievements and challenges of Mg-alloys processing for the improvement of the properties and to meet stringent requirements for medical implant applications. The analysis of the abundant literature unveils the existing knowledge gaps and challenges, of which we highlight the following: (i) For the alloys of a nominally similar chemical composition and comparable microstructure, different researchers report remarkably different results regarding the corrosion rate. (ii) There are only few publications studying the corrosion compatibility of magnesium alloy implants with other metallic implants, which may co-exist in close proximity to each other; for example, permanent dental implants made of titanium alloys may appear next to temporary Mg-based implants, which are widely used in both maxillofacial and musculoskeletal surgery. (iii) Corrosion fatigue has been only scarcely studied, although, in many cases, implants installed in the human body operate under cyclic loads. (iv) A potentially very harmful stress corrosion cracking (SCC) phenomenon occurring in Mg alloys is insufficiently covered so far; this phenomenon manifests itself in the form of sudden failure when the stress level reaches a certain critical value, which is especially important for bioresorbable implants due to the natural increase in the acting stress in the process of their thinning. In the present review, we endeavor to address these little-covered issues, with a focus on the Mg-Zn-Ca system.

## 2. Mechanical Properties of Mg-Zn-Ca Alloys

The contemporary development of biodegradable implant materials is driven by the need for an enhanced mechanical performance while fulfilling the demanding requirements for biocompatibility and control over the rate of bio-corrosion. Several decades of research have shown that the critical physical/chemical properties of magnesium alloys are strongly related to the complex multiscale microstructure of magnesium alloys of grain sizes, crystallographic orientations (texture), and types of grain boundaries. The current research continues previous efforts for tailoring the materials’ functionality through variations in alloy composition, microstructure, and surface treatment. Numerous multidisciplinary studies have convincingly established that mechanical properties [66,67], notably fatigue [68,69] and, concomitantly, the bio-corrosion performance of Mg-alloys [70,71,72,73,74], are strongly favored by grain size reduction, the uniformity of the grain structure, and homogeneity in the distribution of primary and secondary phases. There have been many strategies proposed for refining the coarse as-cast microstructure in wrought Mg-alloys. A variety of these strategies range from the traditional use of chemical agents such as Zr [75] to thermomechanical treatment controlling dynamic recrystallization [76,77,78,79,80], rapid solidification [81], lased additive manufacturing [82], powder metallurgy [18,83], and surface treatment by mechanical attrition [84] or intense ultrasonic treatment [85].

**Figure 1:**
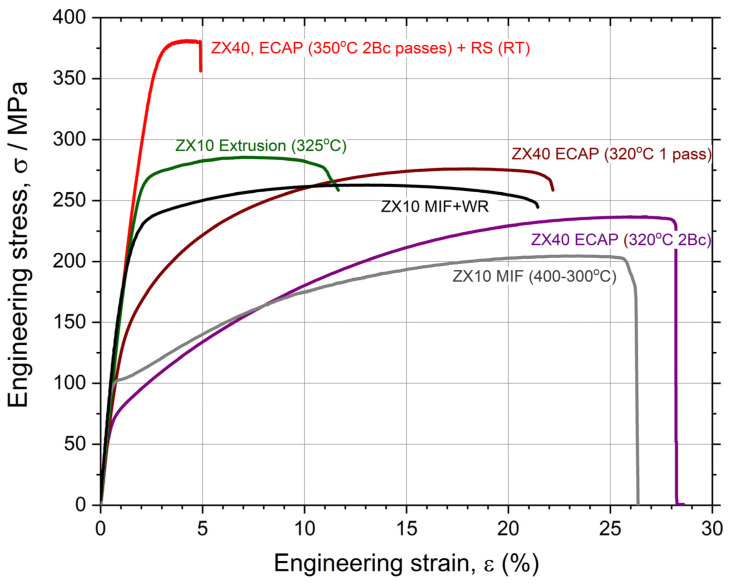
Tesnile stress–strain curves of differently manufactured ZX10 and ZX40 alloys (data are taken from [20] for ZX40 ECAP and ECAP + RS and from [96] for ZX10 MIF and MIF + WR); RT stands for room temperature.

**Figure 2:**
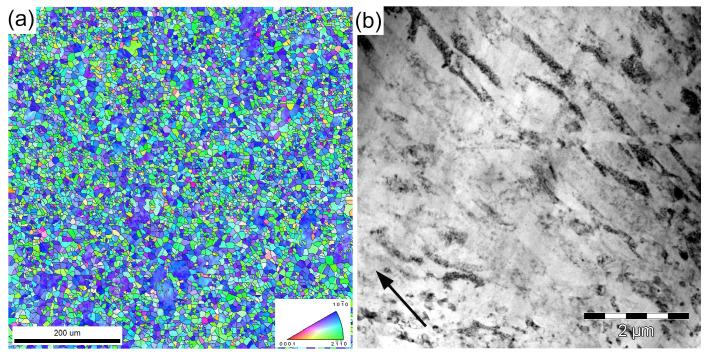
Electron backscattering diffraction (EBSD) orientation map showing the grain structure of the alloy Mg-4Zn-0.16Ca in the cross-section that is normal regarding the extrusion direction after ECAP followed by rotary swaging (**a**) (the inverse pole color code is shown in the inset as a standard stereographic triangle), and the TEM image representing the typical fine grain structure in the longitudinal direction of the same specimen (**b**) (the arrow indicates the extrusion direction); adapted from [96].

To date, extrusion (both direct and indirect) at elevated temperatures and hot rolling are the primary industrially adopted refinement techniques that are widely used to fabricate Mg-based rods and plates with remarkably different properties due to the differently evolving texture in these processing routes. During the conventional thermomechanical processing of wrought magnesium alloys, the microstructure and texture evolve primarily through dynamic recrystallization (DRX). New fine grains generated by DRX feature a low dislocation density and participate in the texture evolution, giving rise to the uniform grain structure (if DRX is completed) with a favorable combination of strength and ductility. Over recent decades, a large group of so-called severe plastic deformation (SPD) methods has emerged as efficient companions to industrial extrusion and rolling. These techniques gained popularity as efficient tools for modifying the microstructure and for strengthening a wide range of metallic materials, including Mg alloys, by imparting extensive plastic strains to a workpiece. Among the wealth of proposed techniques reviewed on many occasions (see, for example, [86,87,88] and the references therein), Equal Channel Angular Pressing (ECAP), pioneered by Segal [89], High Pressure Torsion (HPT), widely known since the early works by Bridgman [90,91], and Multiaxial Isothermal Forging (MIF) [92] are by far the most popular SPD routes for the manufacturing of fine-grain Mg alloys (see a review [67] for details of the Mg processing and outcomes). Remarkable microstructure refinement and the concomitant substantial strengthening effect associated with it are the common results of any of those processing techniques. However, compared to many fcc, bcc, and even hcp materials, the processing of wrought Mg alloys by conventional or SPD techniques has important features associated with (1) specific deformation mechanisms operating in Mg at different temperatures and strain rates [93] and (2) the pivotal significance of DRX for the microstructure evolution in Mg alloys. While there is an obvious trade-off between the tensile strength and ductility, which often appear to be mutually exclusive properties in deformation processing, i.e., an improvement in one is permitted only at the expense of the other [94], wrought Mg alloys deviate from this trend [95]: their ductility benefits strongly from deformation processing to large strains due to a combined effect of grain size reduction, the formation of a favorable crystallographic texture, and the refinement and redistribution of secondary phases [67]. This permits the development of many versatile processing schemes combining the benefits of different techniques. Many researchers confirmed the advantageous effect of such “hybrid” processing. For example, it is reflected in Table 1 and Table 2, as well as in Figure 1, where some results obtained by the present authors regarding Mg-Zn-Ca alloys manufactured by ECAP followed by either rotary swaging (RS) [20] or MIF and warm rolling (WR) [96] are shown. Figure 1 unveils that the considerable ductility (of 25%) gained over the course of initial ECAP processing at elevated temperatures can be “converted” into a high strength through additional strain hardening via either rotary swaging at room temperature or warm rolling. The tensile strength after warm ECAP followed by the cold (room temperature) rotary swaging of Mg-4Zn-0.15Ca can be as high as 380 MPa, which is quite high for this class of nominally low-strength alloys. The microstructure of this remarkably strong alloy is illustrated, for example, in Figure 2 by the electron back scattering diffraction (EBSD) orientation grain map in the transverse direction and the corresponding transmission electron microscopy (TEM) image showing the typical grain microstructure in the longitudinal direction of the ECAP + RS manufactured rod. The distinctive, fully recrystallized homogeneous microstructure with a mean grain size of slightly less than 10 μm and its standard deviation of 5.5 μm is readily observable in Figure 2a. The grains and subgrains tend to elongate in the longitudinal direction (Figure 2b). The strong crystallographic texture paired with the dislocation accumulation during the rotary swaging process was considered responsible for the observed reduction in ductility. Thus, the choice of the processing route can be made by the desire to accentuate either a very high strength (tensile and cyclic) with some compromised ductility or a high ductility with a reasonably high (albeit not ground-breaking) strength.

**Figure 3:**
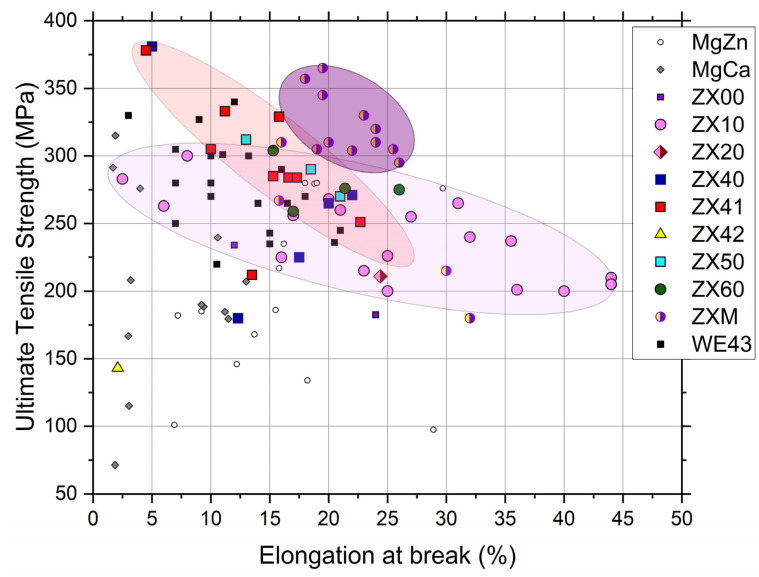
Summary of mechanical properties—strength vs. ductility—of Mg-Zn-Ca alloys with different compositions and microstructures (data are imported from Table 2 and the references therein).

While the overall positive and promising results have been reported for many Mg-based alloys, the results of conventional and SPD processing obtained for the Mg-Zn-Ca system are still limited. Table 2 compiles the results of mechanical testing reported in the literature for differently processed binary Mg-Zn (the effect of Zn concertation), Mg-Ca (the effect of Ca concentration), and Mg-Zn-Ca alloys, with the Zn concentration varying between 1 and 6 wt.%, and Ca varied typically from 0.1 up to 2 wt.%. A very wide range of mechanical properties attainable through different processing routes has been documented, which permits designers to choose the most appropriate deformation processing route, depending on the sought combination of properties—strength and ductility—and economic efficiency. Figure 3 summarizes the available literature data and the original results for various differently produced Mg-Zn-Ca (ZX series) alloys and their popular derivative—Mg-Zn-Ca-Mn (ZXM series) (Table 2)—in terms of ultimate tensile strength, σ_UTS_, plotted vs. elongation to failure in tension, ε_f_. This scatter plot illustrates vividly that the desired properties profile with a wide range of combinations of strength and ductility can be achieved even for lean ZX10 alloys, with the ultimate strength being in excess of 250 MPa, paired with an excellent ductility of 25%. Overall, the mechanical properties of Mg-Zn-Ca alloys are favorably comparable to those of another popular high-strength alloy, WE43 by Magnesium Elektron (Luxfer MEL Technologies, Manchester, UK, since 2018), which is highly alloyed with rare earth elements and has been a potent candidate for temporary implant applications (the data reported in the literature [97,98,99,100] for the alloy WE43 are shown in Figure 3 for comparison). The addition of Mn promotes dynamic recrystallization and grain refinement during thermo-mechanical processing [101]; furthermore, the basal texture sharpens in this way, resulting, finally, in a good balance between the strength and ductility in the ZXM alloy family. Bakhsheshi-Rad et al. [102] have shown that the strength and ductility benefit from the addition of Mn to a tertiary Mg–Zn-Ca alloy due to the enhanced precipitation and grain refinement, and the biocorrosion rate also reduces appreciably due to the improved protection performance of the Mg(OH)_2_ layer incorporating oxidized manganese into the magnesium hydroxide.

**Table 2:** Mechanical properties of Mg-Zn-Ca alloy.

Alloy/Composition	Processing	Grain Size,	Yield Strength,	Tensile Strength, MPa	Elongation	Ref
		μm	MPa		(%)	
Pure Mg	–		27.5	97.5	28.9	[103]
Mg-1.0Zn	As-cast		20	101	6.9	[104]
Mg-2.0Zn			27	146	12.2	
Mg-3.0Zn			47	168	13.7	
Mg-4.0Zn			58	217	15.8	
Mg-5.0Zn			68	185	9.2	
Mg-6.0Zn			69	182	7.2	
Mg-0.4Zn (RD)	Hot rolling	60	93	186	15.5	[105]
Mg-1Zn	As-cast		25.5	134	18.2	[106]
Mg–6Zn	Extrusion			279.5	18.8	[23]
Mg– 6Zn	Extrusion			277–281	18–19.6	[107]
Mg-6Zn	Extrusion		140	280	18	[104]
Mg-0.8Ca	Poly filament					[108]
Mg-0.8Ca	Extrusion			207	13	[107]
Mg-0.8Ca	0.5 mm wire			315	1.9	[107]
Mg-1Ca	As-cast		40	71.38	1.87	[109]
Mg–1Ca	Extrusion			239.6	10.6	[110]
Mg-1Ca	Hot rolling			166.7	3	[109]
Mg–1Ca			72	179.5	11.5	[111]
Mg–1Ca	Extrusion		148	276		[112]
Mg–2Ca			77.2	184.6	11.2	[111]
Mg-2Ca	As-cast		47.3	115.2	3.05	[113]
Mg-4Ca	–		34.5	77.4	53.3	[103]
Mg–5Ca			94.1	188.4	9.4	[111]
Mg–10Ca			109.4	190	9.2	[111]
Mg–15Ca			172.3	208.1	3.2	[111]
Mg–20Ca			234.9	291.3	1.7	[111]
Mg-5.74Zn (Z6)	Extrusion		125	276	29.7	[114]
Mg-6.01Zn-0.36Ca (Zx60)			169	276	21.4	
Mg-6.01Zn-0.82Ca (Zx60)			230	304	15.3	
Mg–4.0Zn–0.2Ca (ZX40)			58.1	225	17.5	[104]
Mg–4.0Zn–0.5Ca (ZX40)			70	180	12.3	
Mg–4.0Zn–1.0Ca (ZX41)			83	175	8.7	
Mg–4.0Zn–1.5Ca (ZX41)			83	167	7.1	
Mg–4.0Zn–2.0Ca (ZX42)			90	143	2.1	
Mg-0.3Zn-0.1Ca (ZX00)	Hot rolling	58	92.5	182.5	24	[105]
Mg-1Zn-0.3Ca (ZX10)	Extrusion	2	240	255	27	[115]
Mg-1Zn-0.3Ca (ZX10)	Extrusion	1.6	238	265	31	[116]
		1.8	247	268	20	
		3	184	240	32	
		6.8	140	226	25	
Mg– 1.2Zn–0.5Ca–0.5Mn (ZXM100)	As-cast		60.3	121.3	3.2	[117]
Mg–1.2Zn–0.5Ca–0.5Mn (ZXM100)	Heat-treated		84.3	150.7	4.9	[117]
Mg-0.21Zn-0.30Ca-0.14Mn (ZXM100)	Extrusion	16.2	125	180	32	[118]
		7.3	165	215	30	
			305	310	20	
Mg-2Zn-2Ca-0.5Mn (ZXM220)	–		78.3	168.5	64.5	[103]
Mg-4Zn-2Ca-0.5Mn (ZXM420)	–		83.1	189.2	69.1	[103]
Mg-4Zn-4Ga	ECAP	5	165	290	22	[119]
Mg-4Zn-4Ga-0.2Ca	ECAP	10	165	255	17	[119]
Mg-4.67Zn-1.27Ca (ZX41)	Extrusion	1	291	329	15.8	[120]
Mg-4.50Zn-1.13Ca (ZX41)	Extrusion		180	212	13.5	[121,122]
			290	305	10	
		12.3	173	251	22.7	
		5.2	228	284	16.6	
		3.8	227	285	15.3	
		3.6	234	284	17.3	
		1.8	320	333	11.2	
		1	370	378	4.5	
Mg-5Zn-0.5Ca	Heat-treated		91	158	4.9	[123]
Mg–1.0Zn	Extrusion	20–50	140	235	16.2	[124]
Mg–1.0Zn–0.2Ca (ZX10)		5–20	140	237	35.5	
Mg–1.0Zn–0.5Ca (ZX10)		6–10	105	210	44	
Mg–1.0Zn–0.3Ca (ZX10)	ECAP	4–8	106	215	23	[125]
Mg–1.0Zn–0.2Ca (ZX10)	ECAP	3.7	90	225	16	[126]
	ECAP + HPT	0.25	220	263	6	
	ECAP + HPT	0.2	230	283	2.5	
Mg-2Zn-0.2Ca (ZX20)	Extrusion		118	211	24.4	[112]
Mg–4.0Zn–0.15Ca (ZX40)	ECAP	28	71	265	20	[20]
Mg–4.0Zn–0.15Ca (ZX40)	ECAP + RS	10	348	381	5	
Mg–4.0Zn–0.56Ca (ZX40)	ECAP	9	127	271	22	
Mg-4.50Zn-1.13Ca (ZX41)	As-cast					
	Extrusion					
Mg-1Zn-0.2Ca (ZX10)	As-cast	185	37	165	22	[96]
	MIF	2.9	100	200	25	
	MIF + WR	2.2	210	260	21	
Mg-1Zn-0.5Ca (ZX10)	As-cast	140–160	55	120	5	[127]
	Extrusion	0.5–0.6	297	300	8	
		2–3	197	256	17	
		5–6	120	200	40	
		8–13	105	205	44	
		20–30	99	201	36	
Mg-5.12Zn-0.32Ca (ZX50)	Extrusion	2	250	312	13	[101]
	ECAP	0.7	230	290	18.5	
Mg-0.95Zn-0.9Ca (ZX00)	Twin-roll casting	7.7	155	234	12	[128]
Mg-5.99Zn-0.98Ca (ZX60)		7.4	164	259	17	
Mg–5.25Zn–0.6Ca (ZX50)	Extrusion		220	270	21	[129]
Mg-5.25Zn-0.6Ca-0.3Mn ZXM(500)			272	305	19	
Mg–6.45Zn–0.2Ca-0.2Mn (ZXM(600)	Extrusion	1.8	290	304	22	[130]
Mg-7Zn-2Ca-0.5Mn (ZXM720)	–		45.4	140.7	82.2	[103]
Mg–6.6Zn–0.19Ca (ZX60)	Extrusion		148	275	26	[127]
Mg–5.7Zn–0.17Ca-0.84Zr (ZXK600)			310	357	18	
Mg-5Zn-0.3Z-0.25Ca-0.1Mn (ZXM500)	Extrusion		330	365	19.5	[131]
			260	320	24	
			305	330	23	
			305	345	19.5	
			210	295	26	
			250	305	25.5	
			260	310	24	
Mg–5.99Zn–1.76Ca–0.35Mn	Extrusion		219	267	15.8	[132]
(ZXM610)			289	310	16	

RD denotes rolling direction.

The analysis of the experimental data presented in Table 2 and Figure 3 reveals several common trends in the development of Mg-Zn-Ca-X alloys. First, the strength tends to increase, on average, with the increased content of alloying elements in a sequence Z→ZX10→ZX40→ZXM. Second, the tradeoff between strength and ductility is also unveiled—the stronger materials tend to have lower ductility. However, the variability of properties following these general trends is high, depending significantly on the processing scheme. The preferred alloy composition and production technique can be chosen on the basis of the required response and economical aspects. Although SPD processing has been proven to be capable of generating an exceptional combination of properties in the working billet, it is obviously not among the most cost-effective methods. It can hardly compete with modern extrusion techniques in large-scale production contexts. However, SPD techniques such as multiaxial forging and rotary swaging can fit the niche for biomedical implant applications where the massive production paradigm shifts towards individual custom-built manufacturing.

## 3. Corrosion Properties: Modern Insights and Challenges

### 3.1. Mg-Zn-Ca in Comparison with Other Compositions

Magnesium and its alloys can corrode through many modes, including uniform corrosion, galvanic corrosion, pitting, filiform, and stress-affected corrosion. The form of corrosion that an Mg alloy suffers from primarily in a specific environment (e.g., saline solution or simulated body fluid) depends on many factors, such as the chemical composition of the alloy and the environment, the alloy microstructure, including the flaws, the local stress risers and dislocations, the crystallographic texture, the grain size, and the shape and distribution of grain boundaries, the impurities, the primary and secondary phases, their morphology, the density, the distribution, etc. Complex multiphase Mg-alloys are highly susceptible to galvanic corrosion caused by the impurity or secondary phases, which act as internal cathodes [4]. Thus, corrosion in Mg alloys often occurs as localized degradation adjacent to the cathode. One strategy for mitigating this effect is to refine the microstructure, which can be achieved through a wealth of deformation techniques briefly outlined in the preceding section. The significance of the corrosion performance of Mg cannot be overvalued, as reflected in multiple excellent reviews, some of which (by far not all) are to be mentioned [12,27,107,110,133,134,135,136,137,138,139,140] (see also the references therein). Since the topic has been extensively covered over the years, in the present communication, we do not intend to duplicate the well-established facts and conclusions regarding the details of the corrosion processes and underlying mechanisms. Instead, we shall focus on the observations of the corrosion rates and related issues.

Corrosion characteristics are of vital importance for bio-degradable temporary implant material since it is the corrosion resistance that determines how long the dissolution of the implant will take in human body fluids and, accordingly, how long the implant can keep its integrity with the healing bone. Table 3 summarizes the corrosion rates for the Mg-Zn-Ca alloys in Hank’s solution, as evaluated by various methods. The results for some other popular alloying systems, such as Mg-Al-Zn and Mg-Zn-Y, are shown for comparison (with words of caution that the precise quantitative comparison is exceptionally challenging due to the differences in experimental conditions). To obtain a more profound impression of the environmental degradation performance, Table 4 shows the corrosion rates of Mg-Zn-Ca alloys in other standard solutions.

Table 3 convincingly shows that the corrosion properties of low-alloy Mg-Zn-Ca materials are on par with those of other bioresorbable magnesium alloys. The corrosion resistance drops significantly with the Zn content exceeding 1–1.5% zinc and Ca exceeding 0.2–0.4%. This is most likely due to an increase in the number of secondary phase particles, which have a more positive electrode potential regarding the magnesium matrix. Table 4 shows that the same trend is also seen in other simulated body fluids (SBFs). Reviewing both tables, one can notice that the results depend not only on the material itself but also, to a great extent, on the experimental conditions and measurement methods used. This is clearly indicated by the fact that the results obtained by the same team of researchers on the same material can differ by several times and even by orders of magnitude, and this remark is true for all the considered alloys (not just for Mg-Zn-Ca). Therefore, in the following sub-section, we shall review the influence of some methodological factors and the measurement methods used to assess the corrosion rate.

### 3.2. Methodological Aspects of Corrosion Tests

#### 3.2.1. Testing Conditions

Human beings are similar and different at the same time. While certain global factors, such as the temperature of body fluids and the pH level, are maintained approximately constant (37 °C and 7.0, respectively), the details of the local chemical composition can be considerably different [157,158]. The chemical composition of the in vitro testing solutions may vary greatly, from a simple physiological solution containing 0.9 wt.% NaCl [159] or Ringer’s solution [160] to complex multicomponent SBFs such as Hank’s [147] and Kokubo’s solutions [161], etc. In common, the bio-corrosion environment contains many chloride ions known as strong corrosive agents for Mg. Different corrosion products can form depending on the composition of both the corrosion environment and the alloy itself. In other words, the chemical composition of the corrosion environment affects the properties of the passivating film formed on the sample and, accordingly, the rate of its dissolution. For example, Hattab et al. [162] revealed that brucite (Mg(OH)_2_) and calcite (M_gx_Ca_1–x_CO_3_) form on the surface of magnesium immersed in Ringer’s solution, while the corrosion product films formed on the Mg surface in Hank’s solution contained additionally calcium phosphate (CaP), which affects the corrosion rate. Similar results were demonstrated by Mena-Morcillo et al. in [142] on the commercial Mg-Al-Zn AZ91 and AZ31 alloys. The authors emphasized that, unlike Ringer’s solution, in Hank’s solution and SBF, the Ca_10_(PO_4_)_6_(OH)_2_ phase was formed beside the magnesium oxide and hydroxide. The corrosion rate in Hank’s solution was found to be significantly lower (from 2 to 8 times, depending on the measurement method and the alloy). This was explained primarily by a more resistant corrosion products layer. Merson et al. examined another popular Mg-Zn-Zr alloy, ZK60, in two similar solutions [163]. It was found that the difference in the corrosion rate between the two solutions was not very large. However, the trend indicated above was maintained: the lower corrosion rate was observed in Hank’s solution, while the materials degraded faster in Ringer’s solution. In the same work, the effect of several other factors, such as the temperature, steering the corrosive medium, and maintaining the pH level, was also considered. One should note that the pH tends to increase significantly (up to 10 or so, depending on the solution [164]) when magnesium is dissolved in water or aqueous salt solutions due to an increase in the concentration of OH-ions. The pH value exerts a tremendous effect on the corrosion rate. Thus, maintaining the pH of a laboratory corrosion solution at a physiological 7.0 ± 0.2 level is an essential experimental condition for simulating the natural environment of a human body. The pH level can be maintained in different ways. The partial replacement of the medium was used in [144,163]: when a certain pH level was reached, the solution was evacuated from the cell, and the fresh solution was supplied. The procedure was fully automated through the use of a digital pH meter, an electronic controller, and a dual-head peristaltic pump. Alternatively, the replacement of the corrosive medium could be carried out manually [165,166]. Unlike many reports, Bornapour et al. [166] concluded that the renewal of the environment had a negligible effect on the corrosion rate of the Mg-Sr-Ca alloy.

An alternative method of pH correction, which is widely adopted in laboratory practice, is the use of buffering systems to regulate the pH value of the SBF to the near-neutral condition [4]. Dilute acid solutions are most commonly used for this purpose: HEPES, Tris–HCl, and HCO_3^−^_/CO_2_. The authors underlined that, although all these agents significantly affect the corrosion of Mg alloys, HCO_3^−^_/CO_2_ is admittedly the most important buffering system in the human body: not only is it capable of consuming the OH^−^ ions, but it also induces the precipitation of relatively stable compounds such as MgCO_3_ that enhance corrosion protection. This is further demonstrated by Xin et al. in [167], where the influence of the concentration of the HCO_3^−^_ ions on the corrosion of magnesium was studied. The results obtained showed that an increase in the concentration of the bicarbonate ions in SBF from 4 to 27 mmol/L reduced the corrosion rate by almost a factor of ten.

The present authors developed a fully automated PC-controlled system to maintain the pH level at a desired value [96,156]. Considering the above-mentioned features of the formation of calcium compounds with phosphorus in a solution containing the phosphate ions (for example, Hank’s solution), it can be assumed that pH correction can affect the formation of corrosion products and, hence, the resultant corrosion properties. Since many compounds of calcium and phosphorus (e.g., orthophosphate and hydroxyapatites), as well as magnesium and calcium carbonates, are poorly soluble, this factor should be taken into account when developing the Mg-Zn-Ca alloy testing methodology, particularly when choosing a pH correction method.

A consensus on how to properly maintain the pH level has not yet been reached. Moreover, the pH level is sometimes used as an indirect indicator characterizing the course of corrosion processes. In this way, no pH adjustment is acceptable in principle. The significance of pH correction for the reliability of the corrosion rate measurements is hard to quantify, in general, since the above examples show conflicting results. This is likely to be highly dependent on the composition of the corrosive medium used and the chemical composition of the alloy. It should be noted that the pH level during the experiment does not necessarily increase monotonically. Instead, it can first increase and then decrease, as shown by Li et al. [168] and Bazhenov et al. [119]. Li and co-authors attribute the decrease in pH to the onset of a chemical reaction to form apatite, which consumes hydroxide ions from the solution, thereby lowering the pH. Since hydroxyapatite formation also occurs with the consumption of calcium ions, when dealing with Mg-Zn-Ca alloys, one should be aware of the possibility of a reaction occurring, reducing the pH level. Bazhenov et al. suggested that some microorganisms develop in a corrosive environment, whose vital activity is capable of influencing the pH level. In both cases, Hank’s solution was used, which contains both calcium ions and phosphate groups involved in the formation of hydroxyapatite and a large amount of glucose, which makes the corrosive medium quite nutritious for microorganisms, so it is possible that both of the abovementioned processes can be the cause of the observed drop in the pH. The fact that the change in the pH depends on several independent factors makes the pH indicator unreliable for assessing the intensity of corrosion processes. Moreover, after reaching a certain value, the pH tends to saturate or even reduces slightly before reaching a steady value. Regardless of this change in the pH value, the corrosion processes continue to evolve and can even become more intense [155,169]. Therefore, more accurate and reliable methods should be used to determine the critical corrosion characteristics.

#### 3.2.2. Methods for Determining the Corrosion Rate

The corrosion rate of magnesium alloys can be assessed by different means. The most popular methods are weight loss, hydrogen evolution and a family of electrochemical techniques. Gravimetric measurements are simple and well-established: the specimen is weighted before and after corrosion testing. Corrosion products are chemically removed from the corroded surface using standard solutions, the recipes of which are given in ASTM G1-03 [170]. For the Mg-Zn-Ca alloys, the water solution of CrO_3_ [145,146,171,172] is most commonly used. The solution of CrO_3_ (from 18% [148] to 20% [173]) with the addition of AgNO_3_ (from 0.1% [148] to 1% [173]) is also proven to be efficient, particularly if the cleaning is performed in an ultrasonic bath. The corrosion rate is calculated according to the ASTM G31 (NACE TM0169) standard [174]. The key advantages of the gravimetric technique are its simplicity and applicability to samples of virtually any shape and dimension. The disadvantage is, however, the inevitable inaccuracies resulting from the removal of corrosion products, giving rise to either the overestimation or underestimation of the corrosion rate as a consequence of insufficient or excessive cleaning after immersion, respectively [175]. Furthermore, weight loss measurements provide only an average corrosion rate and do not allow for following the change in the rate of corrosion during immersion into a corrosive environment.

The latter limitation is alleviated by using the hydrogen evolution method. During the corrosion of magnesium in aqueous salt solutions, the following reactions occur [176,177]:

2H^+^ + 2*e*^−^ = H_2_↑ (cathodic partial reaction)

2Mg = 2Mg^+^ + *e^−^* (anodic partial reaction)

2Mg^+^ + 2H_2_O = 2Mg^++^ + 2OH^−^ + H_2_ ↑ (chemical reaction)

2Mg + 2H^+^ + 2H_2_O = 2Mg^++^ + 2OH^−^ + 2H_2_ ↑ (overall reaction)

Mg^2+^ + 2OH^−^ = Mg(OH)_2_ (product formation)

Mg + 2H_2_O = Mg(OH)_2_ + H_2_ ↑ (overall reaction in the steady state)

Thus, the evolution of one mole of hydrogen gas corresponds to the dissolution of one mole of magnesium metal. For volumetric hydrogen evolution collection measurements, an inverted funnel attached to an upside-down burette immersed in a corrosive solution specimen is most commonly used [178]. The method, in addition to the obvious advantages, has some annoying shortcomings. In particular, hydrogen evolution allows for determining only the amount of the loss of material that has reacted chemically with the release of hydrogen, while the particles that have detached from the sample due to significant corrosion damage [169] or the particles of chemically stable secondary phases, such as the LPSO phase [144,179], are not taken into account.

**Figure 4:**
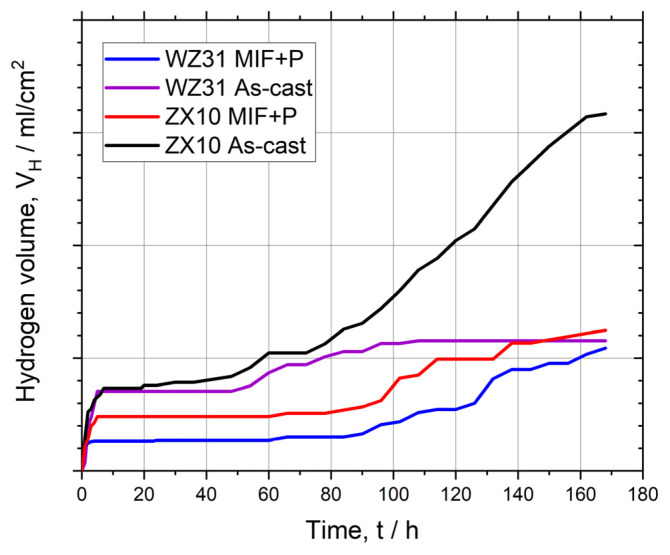
Hydrogen evolution plots in WZ31 and ZX10 alloys with different microstructures in Hank’s solution thermostabilized at 37 °C and maintained at pH = 7.0 (adapted from [144]).

Using the hydrogen evolution method, a graph showing the change in the corrosion rate over time can be produced, which is very insightful for the overall assessment of the biodegradability of Mg alloys. The significance of such information is hard to overvalue due to the considerable variability of the corrosion rate in magnesium alloys over time. Corrosion processes can accelerate, decelerate, or proceed steadily at a constant rate. Moreover, the corrosion rate can change its trend several times during the dissolution of the implant. Figure 4 exemplifies the corrosion processes assessed by hydrogen evolution in the alloys Mg-Zn-Ca and Mg-Y-Zn in different conditions—as-cast and after the multiaxial isothermal forging (MIF) followed by upsetting [144]. The kinetics of the corrosion processes occurring in these alloy systems over 168 h in Hank’s solution are clearly traced. It can be seen that, during the first 6 h, corrosion is most intensive. Its rate then drops sharply. The transition point corresponds to the formation of a dense passivating film on the surface. As long as the integrity of the passive layer is not compromised, the corrosion rate is relatively low and steady. This stage is reflected by a plateau on the graph. After the local destruction of the film of corrosion products, localized corrosion starts to develop. The rate of this process bounces up accordingly, and the hydrogen evolution trends for the ZX10 and WZ31 alloys after SPD turn upwards. The corrosion kinetics of as-cast WZ31 are remarkably different. This alloy is not prone to intense localized corrosion in Hank’s solution. Therefore, after passivation, its corrosion rate is constant. This fact highlights another little-covered aspect, i.e., the duration of in vitro corrosion tests of bioresorbable magnesium alloys should be sufficient to disclose existing stages of surface degradation.

Electrochemical methods—potentiodynamic polarization (PDP) [180] and electron impedance spectroscopy (EIS)—are important tools that are widely used to understand the kinetics of the early stages of corrosion. They are also popular means for the quantitative characterization of the corrosion rate. Using the PDP method with Tafel extrapolation for measuring the corrosion rate *P_i_* (mm/yr), the corrosion current density, *i_corr_* (mA/cm^2^), is estimated by extrapolating the cathodic and anodic branches of the polarization curve, and *i_corr_* is related to the average corrosion rate using:

(1) $$P_{i}{}_{}=22.85\;i_{corr}$$

The polarization resistance $R_{P}$ can be estimated using the Stern–Geary ratio:

(2) $$R_{P}=\frac{1}{i_{corr}}\frac{\beta_{a}\beta_{c}}{2.303\left(\beta_{a}+\beta_{c}\right)}\;$$

with $\beta_{a}$ and $\beta_{c}$ being the slopes of the anodic and cathodic branches of the Tafel plot.

EIS is an electrochemical technique which uses a small sinusoidal electric stimulus (potential or current) to measure the volt-ampere response and calculate the impedance at different frequencies. EIS is capable of detecting slight changes in the electrode surface state and correlating electrochemical processes at the electrode/electrolyte interface without changing the surface state. Due to its intrinsic features, EIS is widely used to study the corrosion behavior and corrosion mechanism of various uncoated and coated metals. Both methods are well established and standardized. The detailed procedure for the corrosion rate calculation is described in the ASTM G102-89 standard [181].

The electrochemical methods have become widespread due to their ability to deliver quick estimates of corrosion rates. At the same time, this is a primary shortcoming of these methods—they measure the corrosion rate too rapidly. Many researchers question the reliability of the data obtained. For example, Atrens et al. [136] presented a comprehensive analysis of the corrosion rates assessed by different methods: electrochemical (PDP or EIS), hydrogen evolution, and weight loss. The results obtained by the hydrogen evolution and the gravimetric method are usually reasonably close. On the other hand, the results obtained from electrochemical methods on the same materials are often underestimated, sometimes by orders of magnitude. Two main reasons for this discrepancy can be proposed. First, the electrochemical measurements are commonly carried out almost immediately after the submersion of the sample into an electrolyte. The initial corrosion rate may differ significantly from that observed in long-term immersion tests due to the chemical evolution of the corrosion film on the metal surface. Second, a significant effect can be anticipated from hydrogen bubbles profusely formed on the surface of the sample and from isolating it from the corrosive medium. For magnesium alloys, electrochemical methods can also significantly overestimate the corrosion rate. For example, the corrosion rate measured by the electrochemical methods in ref. [119] was found to be 6–10 times higher than the values obtained from hydrogen evolution during long-term exposure in an aggressive environment. [45] A comparison of the corrosion rates obtained from short-term (3 days) and long-term (30 days) immersion tests with the results derived from electrochemical measurements has been made for pure magnesium and Mg-6Zn alloy [104]. Both materials exhibited approximately the same trend: the corrosion rate measured by electrochemical methods was significantly lower than that obtained with short-term exposure to the environment but markedly higher than the results of long-term tests. All these findings indicate that the reliability of data obtained by electrochemical methods is questionable, and the weigh-loss and hydrogen evolution methods remain the “golden standards” for the practical assessment of the corrosion rates of magnesium alloys.

One should bear in mind the well-known limitations of the Tafel extrapolation method, which may affect the estimates of the corrosion resistance. Not only should the branches of the polarization curves be well defined and steady, but, perhaps most importantly for Mg alloys, the corrosion should be general in nature, and localized corrosion should not occur. During general corrosion, the electrochemical reactions proceed uniformly over the entire exposed metal surface over a large area, which is rarely observed in Mg alloys (see the review by Kirkland at al. [134]) due to the only partial surface protection by the film of corrosion products [182]. In the author’s experience with the Mg-Zn-Ca system of interest, uniform corrosion has never been observed, as will be further discussed below.

**Figure 5:**
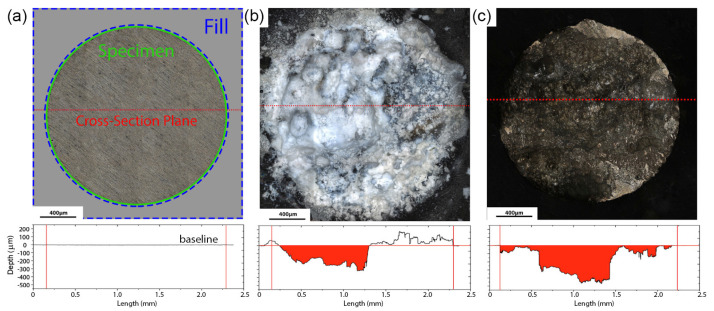
Panoramic images of the sample surface obtained using CLSM and methods for determining the volume of metal lost as a result of corrosion: before corrosion tests (**a**), after holding in Ringer’s solution (**b**), after removal of corrosion products (**c**) [183].

An interesting method for determining the corrosion rate of magnesium alloys has been proposed in [183], where corrosion tests were carried out on samples prepared as metallographic sections embedded in an inert polymer, leaving only one side exposed to the environment. After the test, the samples were extracted from the solution and the corrosion products were chemically cleaned out. Then, the surface was scanned by a confocal laser scanning microscope (CLSM), and the 3D-height map of the corroded surface was obtained. Using the intact polymer plane as a reference, the volume of the corrosion-degraded material can be accurately calculated from the obtained height maps, as illustrated schematically in Figure 5.

The advantage of the proposed technique is that, besides the integral assessment of the overall corrosion rate by the volume of the lost metal, i.e., the data similar to those gained from the conventional gravimetric or hydrogen evolution methods, it allows for examining the local propagation of corrosion damage, concluding on the severity of localized degradation and assessing the mechanism of corrosion. The metrological aspects of the method have been described in detail sufficient for reproduction, thus leading to an easy comparison of the results obtained in different laboratories. The influence of the sample size, geometry, and preparation are discussed, and the necessary guidelines are provided to ensure the reproducibility of the results. Comparing the proposed method with weight loss renders similar results for the overall corrosion rate estimated by both methods, with a difference of only 7%, while the scatter of corrosion rates measured by either of these methods on a series of identical specimens was 10%. Thus, the sufficiently high accuracy and reliability of the proposed method have been demonstrated. One should reiterate that none of the methods considered above, except for the latter one, make it possible to evaluate the local corrosion rate, which can be, by orders of magnitude, higher than the uniform corrosion rate. For example, in the considered study, the maximum local corrosion rate determined by the CLSM technique was found to be four times higher than the average overall corrosion rate. This can be a severe issue for practitioners and implant designers relying on the average corrosion rate documented on the basis of either weight loss, hydrogen evolution, or electrochemical methods for magnesium alloys [96,161]. The only disadvantage of the profilometry-based method is seen in the need for specialized equipment such as CLSM or other comparable profilers, e.g., white light interferometers, etc. It is also worth noting that the molding of the polyethene compound is carried out at elevated temperatures and pressures, which can affect the structure of thermally sensitive and easily deformable Mg-alloys. This is, however, not an issue for many wrought Mg-alloys, including Mg-Zn-Ca, deformed at elevated temperatures and exhibiting notably high yield stresses.

Of particular interest is the fact that the CLSM-based method can be easily applied to magnesium single crystals with different crystallographic planes exposed to the corrosive environment. This opens up a way to examine the long-standing issue of the texture-related anisotropy of the corrosion properties in magnesium.

### 3.3. Anisotropy of Corrosion Behavior

The anisotropy of corrosion properties and the directionality of corrosion propagation are frequently reported for magnesium and its alloys, including Mg-Zn-Ca. There may be several reasons for these phenomena. The first and most obvious reason is associated with the above-mentioned crystallographic factor. It is well-known that the corrosion of magnesium proceeds more intensively on grains of a certain orientation. In one of the early works [184] performed on magnesium single crystals, it was found that, in a chlorine-containing medium (aqueous solution of 0.01 M NaCl_2_ with the addition of dichromate), the basal plane (0001) exhibited the lowest resistance to corrosion. Corrosion itself occurred through a specific “filamentary-like” (filiform) damage, which propagated along a certain direction, not randomly, and this direction is determined by the crystallographic orientation. Conflicting results have been reported in [185], where the (0001) plane in Mg single crystals showed a higher resistance to corrosion. A coarse-grain magnesium polycrystal with the well-characterized orientation of individual grains exposed to a corrosion solution was employed in ref. [186]. The anisotropy of corrosion properties was also recorded, depending on the crystallographic grain orientation: the (0001) orientation showed the highest resistance to corrosion, similar to the results reported in [185]. In a fine-grained Mg, the orientation of individual grains did not have a noticeable effect. However, the integral effect of the crystallographic texture was still appreciable. While the strong effect of the texture on the mechanical properties of magnesium alloys has been well established and understood, the influence of the crystallographic texture on the corrosion properties is less clear. In ref. [187], corrosion tests of commercially pure extruded magnesium with a grain size of 20 μm were carried out using the PDP method. The samples were cut in three different directions—along the extrusion direction, normal in relation to it, and at 45° in relation to it. Samples cut along the extrusion axis showed the lowest corrosion rate, while those with the normal orientation degraded most rapidly. This observation was explained by the presence of a large number of grains with the (0001) orientation in the longitudinally cut samples. Overall, it is safe to say that crystallography does exert a strong influence on corrosion properties, and this effect is manifested in both single and polycrystals with fine and coarse grains. However, the significance of the basal plane in corrosion resistance remains questionable. Gerashi et al. [188] explain these contradicting results through the features of passive film formation. These features strongly depend on external conditions: the aggressiveness of the medium, the presence of passivating agents such as chromate or dichromate in the solution, etc. For example, it was pointed out that the presence of active passivating elements in the solution leads to an increase in the risk of filiform corrosion. All this suggests that the experimental conditions can influence the result to a greater extent than the characteristics of the material itself. Therefore, when studying the effect of the crystallographic orientation on the corrosion properties of magnesium alloys for biomedical applications, it is important to simulate their operating conditions under various scenarios as closely as possible. In particular, for the bio-corrosion-pertinent studies in general, it is essential to use solutions similar in composition to human body fluids containing amino acids, vitamins, and essential proteins, which are known to affect the corrosion behavior significantly [3]. Notably, proteins such as albumin act as effective inhibitors of bio-corrosion, which slows down considerably (up to a factor of 100) when proteins are present in the corrosion medium [189]. This effect is explained as protein adsorption on the surface forming a relatively stable corrosion-resistant layer [190]. In line with the discussion in the previous section, electrochemical measurements, while being helpful for investigating the early stages of the corrosion reactions and passivation processes, should not be used alone for a realistic assessment of the corrosion performance in either in vitro or in vivo conditions. The same conclusion has been drawn in [191], where the influence of the grain size and crystallographic texture of the Mg-0.7Zn-0.6Ca alloy on its corrosion properties was investigated. In the same work, the authors highlighted the significance of intermetallic particles of secondary phases; for Mg-Zn-Ca alloys, this is primarily the Mg_2_Ca intermetallic compound. As a general note, it has been well established that particles of secondary phases have a huge impact, usually detrimental, on the extent of galvanic corrosion and the overall corrosion resistance of magnesium alloys. The physical reason is simple: magnesium has the most negative electrode potential of all structural metals, so the inclusions of almost any secondary phase provoke intense galvanic corrosion. As a result, the magnesium matrix dissolves rapidly. The Mg_2_Ca intermetallic compound is somewhat unusual in this regard—it is an anode in relation to the magnesium matrix, which means that it dissolves itself, first of all. This phase is formed mainly along the grain boundaries. It is therefore plausible to suppose that, in the case of an elongated grain shape, which is often seen after rolling or extrusion, this phase will provide an easy corrosion path, thereby explaining the observed directionality of the corrosion behavior. The role of the corrosion products formed as a result of the dissolution of this phase is also not entirely clear. It has already been mentioned that many calcium compounds are poorly soluble in water and are quite stable; for example, orthophosphate and hydroxyapatite are even actively used in scientific research as protective coatings on magnesium alloys for medical purposes [55,161].

Using atomic force microscopy, potential distribution maps were obtained from the surface of the Mg-1Ca alloy [192]. It was demonstrated that, although the Mg_2_Ca phase does have a more negative potential compared to the Mg matrix in Ringer’s solution, it can nevertheless form corrosion products whose potential is more positive than that of magnesium. In other words, by dissolving itself, the Mg_2_Ca phase produces a substance that leads to the accelerated dissolution of the matrix. This gives rise to the enhanced passivation of the magnesium matrix near the secondary Mg_2_Ca phase under corrosion. However, there is a risk that the higher volume fraction of this phase can adversely affect the corrosion rate. For example, an increase in the corrosion rate was observed in the Mg-2Zn-XCa alloy (X = 0, 0.2, 0.4, 0.8% wt.) with the increasing calcium content and the corresponding volume fraction of the Mg_2_Ca phase [146]. However, distinct peaks of the Mg_2_Ca phase appear in the X-ray diffraction pattern only at 0.8% Ca concentration. The alloy with this amount of calcium also demonstrates a pronounced increase in the corrosion rate: while the increase in the corrosion rate as the calcium concentration increased from 0.2% to 0.4% was only ~1.5 times, the corrosion rate of the alloy with 0.8% Ca was four times higher than that in the alloy with 0.2% Ca. It has been indicated that other intermetallic compounds are formed instead of Mg_2_Ca in Mg-Zn-Ca alloys at specific zinc contents, ultimately affecting the overall corrosion resistance [172,193]. X-ray diffraction analysis showed that, as the Zn content exceeded 1%, the Ca_2_Mg_6_Zn_3_ phase was formed instead of Mg_2_Ca [146]. The dependence of the corrosion rate of the Mg-XZn-0.2Ca alloy on the zinc content shows that the corrosion rate decreases with the increase in the zinc content from 0% to 1%; it then turns to a considerable rise with the further increase in the Zn concentration. This is likely associated with the phase composition change: when 1% Zn is introduced, the Mg_2_Ca phase disappears; however, a further increase in Zn provokes the appearance of the Ca_2_Mg_6_Zn_3_ intermetallic compound, which has a powerfully negative effect on the corrosion properties of Mg–Zn–Ca alloys [194].

**Figure 6:**
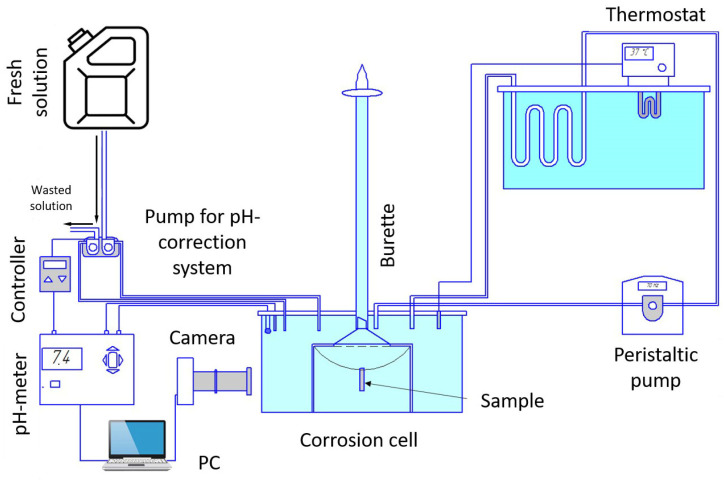
Immersion test setup with automated pH adjustment and hydrogen storage measurement, controlled circulation of the corrosive media, thermal stabilization, and a video observation system.

To illustrate the significance of the microstructure-related anisotropy of corrosion processes, we performed experiments using the alloy ZX10 as follows. A sample of 20 × 10 mm^2^ and a thickness of 0.2 mm was cut from a rolled material with the long side aligned with the rolling direction. To hang the plate in the electrolyte bath, a hole of Ø1 mm was drilled near its short side. After careful cleaning with HF, the plate was hung in a corrosive medium (an aqueous solution of 0.9% (wt.) NaCl) using a fiberglass thread. A glass cell has a capacity of 5 L. The specimen surface was observed by the optical digital video camera. The temperature of the solution was maintained at 37 ± 1 °C, and the pH level was kept at 7.4 ± 1 using the automatic partial replacement of the corrosive media. Figure 6 illustrates the setup.

**Figure 7:**
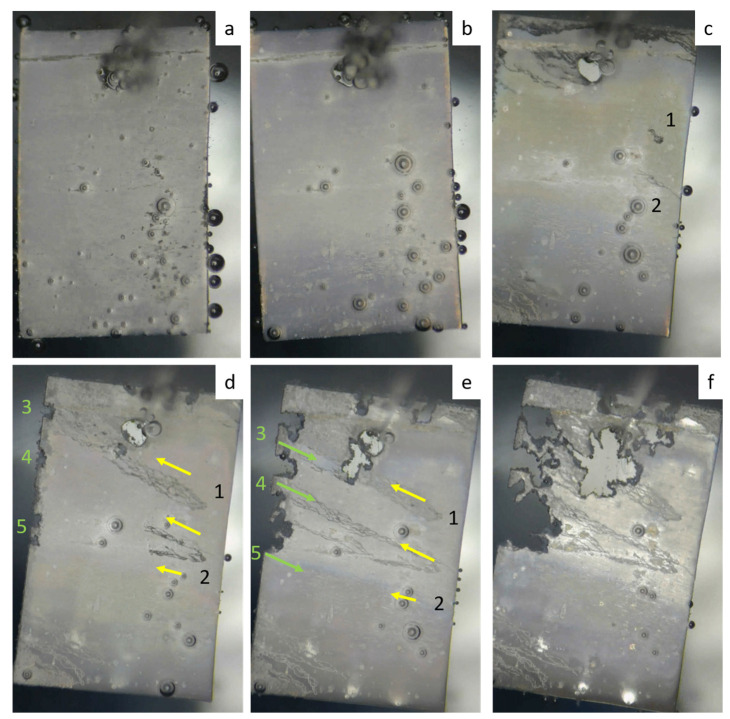
Evolution of the morphology of corrosion damage in the ZX10 alloy ((**a**)—after 30 min of the immersion test, (**b**)—after 12 h, (**c**)—after 24 h, (**d**)—after 36 h, (**e**)—after 48 h, (**f**)—after 72 h).

Figure 7 shows the images illustrating the stages of the propagation of corrosion damage on the sample surface. During the first 12 h, no visible changes occur, except for the release of hydrogen bubbles (Figure 7a,b). After 24 h, the first sites of filiform-like corrosion become visible on the surface (labelled as 1 and 2 in Figure 7c, respectively). After 36 h, these first foci spread over a large surface area in the form of a characteristic river pattern. Furthermore, one can see that these patterns extend in the same direction, which does not correspond to the rolling direction (yellow arrows in Figure 7d). At the same time, large foci of local corrosion are formed at the end surface (labelled as 3, 4, and 5 in Figure 7c). While filiform corrosion is confined only to the surface layer, the localized pitting corrosion affects the entire thickness and propagates through. It is interesting that the initially intense degradation, which nucleated at the local corrosion centers 3, 4, and 5, does not affect the regions of filiform corrosion and propagates mainly in the opposite direction (green arrows in Figure 7e). A similar-in-appearance type of corrosion damage occurs near the hole. However, its growth occurs in the direction of rolling. After 72 h from the beginning of the experiment, the corrosion damage only tends to increase without changing the established trends.

From the above example, it follows that the anisotropy of corrosion properties and the directionality of the corrosion process manifest themselves through at least two corrosion modes: filiform surface corrosion (centers 1 and 2, Figure 7) and local pitting corrosion (centers 3, 4, and 5, Figure 7). Despite the fact that there is no clear understanding of the causes and mechanisms of such behavior at the moment, this example vividly demonstrates the anisotropy of corrosion properties in Mg-Zn-Ca alloys, which is extremely important to take into account when developing medical products from them.

### 3.4. Corrosion Compatibility

As has been noticed above, almost any alloying elements adversely affect the corrosion rate due to the promoted galvanic corrosion. It is therefore plausible to anticipate that the proximity of “foreign” metals to magnesium implant alloys can have a detrimental effect and accelerate the dissolution process of the Mg-based part. In medical practice, particularly in maxillofacial surgery, the probability of deploying a new temporary implant near the existing metallic components used in dental orthopedic permanent structures is relatively high. The compatibility issue with other materials therefore has to be well understood and documented. The base metal alloy systems most commonly used for both temporary and long-term restoration and implant applications in dentistry today include titanium and its alloys, gold, stainless steels, nickel-chromium, and cobalt-chromium alloys. Additionally, a wide range of metals in the form of an amalgam is still used for fillings, and these metals include silver, tin, zinc, and copper. Despite the urgent need to address this topic, relevant publications are still scarce. As early as 1932, Lambotte reported on a surgery strategy that was based on the fixation of a pure magnesium plate into the tibia with six steel screws. It was noted that, already a day after the operation, the patient developed extensive subcutaneous gas cavities, local swelling, and pain, due to which the implant had to be removed after a week [195]. However, a positive note was made in [196], where a titanium implant was attached to a bone with titanium screws, and, additionally, magnesium screws were used at the fracture site. The authors pointed out that the magnesium screw not only provided sufficient fixation of the fracture but also stimulated more intensive healing by increasing the callus and facilitating its mineralization. However, the dissolution rate of the magnesium screw was not evaluated in this work.

An in vivo and in vitro study by Hou, P. et al. [197] showed that pure magnesium plates connected directly to titanium specimens suffered from more significant degradation compared to a reference group of magnesium specimens. In addition, it was noticed that enhanced corrosion occurs even when dissimilar plates are not in direct contact. It was observed that the effect of titanium proximity on the corrosion behavior of magnesium plates decreases as the distance increases between Ti–Mg neighbors. A distance of 5 mm or less between pure Mg and Ti plates was found to be critical for the faster degradation of a magnesium sample. Similar results were obtained in in vivo trials on rats. The metal regions that were in immediate contact with blood vessels degraded most seriously. That is, the blood flow acted as a “conductor” between dissimilar screws, forming galvanic cells and promoting the deterioration processes.

To the best of our knowledge, the only study on the corrosion compatibility of Mg-Zn-Ca ZX10 (Mg-1Zn-0.16Ca) and titanium alloys has been reported in ref. [155]. The influence of the distance between the titanium implant and the Mg alloy plate on the corrosion rate of ZX10 was investigated. The ultrafine-grained ZX10 alloy was manufactured by the SPD MIF process, followed by hot pressing. The tests were carried out in a circulating physiological solution using the experimental setup illustrated in Figure 6 under conditions similar to those described above at 37 ± 1 °C. During the corrosion testing, the titanium implant was placed 3, 6, and 12 cm apart from the ZX10 sample. Additionally, the samples from the reference group were tested without a titanium implant. A galvanic effect detrimental to the corrosion performance was clearly observed at the shortest distance of 3 cm between the test pieces. The corrosion rate was 1.5 times higher compared to that of the reference group. Furthermore, the corrosion damage was much more extensive. From a distance of 6 cm, the titanium implant did not cause any visible effect on the corrosion of the magnesium sample. Thus, the effect of the acceleration of corrosion processes is observed with the simultaneous presence of the magnesium alloy and other metal parts in the same biologically active medium. This must be taken into account in both practical surgery guidelines when the Mg-based alloys are used as temporary structures and in laboratory in vitro testing, e.g., fatigue, which will be discussed in what follows.

## 4. Environmentally Affected Mechanical Response

Temporary implants are load-bearing components. For example, screws, rods, and plates used for the treatment of bone fractures experience a static load due to screw tightening and the misalignment of the bones parts to be fixed, while the regular physical activity of the patient, such as walking, chewing, etc., causes low-cyclic loading. The cardiovascular stents reinforcing the weakened walls of a coronary artery undergo high cycle loading due to heartbeat contractions. Furthermore, the mechanical stress from the external loads can be supplemented by the residual internal stress induced in the metal of an implant by the manufacturing process or surgery procedures, e.g., the bending of the fixating plates. The strength of Mg alloys degrades rapidly as corrosion proceeds. Zhang et al. [104] reported that the bending strength of Mg-6Zn dropped by a factor of 1.6 after a weight loss of only 6% in a physiological saline 0.9% NaCl solution. The collective action of a corrosive environment and mechanical stress (external and/or internal) is capable of inducing stress-corrosion cracking (SCC) or corrosion fatigue (CF) in the metal, depending on whether the static or cyclic stress is applied. Therefore, particular attention should be paid to the SCC and CF of temporary metallic implants because even relatively low service stress in the presence of aggressive environments such as natural body fluids can cause sudden brittle fractures of the metallic component before the completion of a healing process. Indeed, the in-service SCC- and CF-related failures of traditional permanent implants made of stainless steels and Ti alloys have been frequently documented [198,199,200,201]. Although accidents of this kind have not been reported for biodegradable Mg implants so far, the problem of their SCC and CF resistance is seen to be even more acute than that for permanent ones. First, this is due to the much higher susceptibility to the corrosion and the SCC of Mg alloys compared to Ti and stainless steels. Second, it is because the reduction in an implant’s body due to biodegradation can result in a rise in stress during the healing process if the bearing capacity of the growing tissues recovers insufficiently quickly. Furthermore, due to the great propensity of the vast majority of Mg alloys to localized corrosion, the stress in the local regions ahead of the deep corrosion pits can be substantially higher than the average applied stress. Since such stress risers develop very rapidly in the aggressive solutions and serve as favorable sites for SCC, in fact, uncontrollable premature failure due to SCC can occur before the regeneration of the damaged tissues has been completed. Thus, the Mg alloys considered for biomedical applications must possess an excellent resistance to SCC, CF, and corrosion, particularly the localized one. Despite the high importance of the issue, the available data on the SCC and CF of biodegradable Mg alloys, especially of the Mg-Zn-Ca system, are still quite limited. Furthermore, similar to the situation with corrosion rate testing, due to the lack of standards regulating the procedure of the SCC and CF testing of biodegradable alloys, the data provided in available reports were obtained in different experimental conditions, including the strain rate, the chemical composition of the corrosion solution, the temperature, the pH level, etc. So, the effect of the testing conditions on the results of SCC and CF testing is another aspect of particular interest, which was, however, just scarcely studied.

### 4.1. Stress Corrosion Cracking in Mg Alloys

Mg alloys are known to be extremely prone to SCC in various corrosive environments, including distilled water [202,203,204], saline solutions [205,206], and all simulated body fluids (SBF) [163,202] commonly used for the examination of the corrosion properties of biomedical Mg alloys. The susceptibility of the alloys to SCC can be characterized by a number of characteristics, including the ultimate tensile stress and elongation to failure in a corrosive medium, the threshold stress (σ_SCC_) and threshold stress intensity factor (K_1SCC_), below which SCC does not occur, as well as by a few variations in the SCC susceptibility indexes (I_SCC_), which are equal to the loss of the ductility or strength (or both) of an alloy in a corrosive environment with respect to the alloy tested in air. The available data on σ_SCC_ and K_1SCC_ for biodegradable alloys are still rare because of the complexity of the crack growth testing in corrosive environments. Thus, most commonly, the SCC of Mg alloys is assessed by the mechanical properties and I_SCC_ indexes obtained through the uniaxial slow-strain rate tensile (SSRT) testing of the round-shape or flat specimens being exposed to corrosive media and ambient air.

Only two papers have been published so far that shed light on the SCC behavior of Mg-Zn-Ca alloys. Choudhary et al. studied the SCC of three extruded Al-free alloys, including WZ21, WE43, and ZX50, having 7, 15, and 4 µm average grain sizes, respectively [207]. All the alloys were tensile-tested in an m-SBF (modified simulated body fluid) solution with a 3.1 × 10^−7^ s^−1^ strain rate. It was found that the ZX50 alloy demonstrates superior strength in air (352 ± 17.7) as well as in a corrosive environment (257.0 ± 5.6 MPa). However, in terms of the elongation to failure in m-SBF and the ductility loss index, ZX50 showed the worst SCC resistance, which was attributed to the high susceptibility of this alloy to pitting/localized corrosion in m-SBF. The fractographic analysis showed that the SCC in the ZX50 alloy extended through the transgranular mode from the multiple initiation points associated with corrosion pits on the specimen side surface. Jafari et al. [208] investigated the effect of the extrusion temperature on the SCC resistance of the alloy ZX10 using SSRT testing at 3.1 × 10^−7^ s^−1^ in the m-SBF solution. It was established that the alloy obtained by extrusion at 325 °C demonstrated a finer average grain size (1.2 ± 0.8 μm) and higher strength and ductility both in air and m-SBF with respect to those of the alloy extruded at 400 ºC. It is worth noting that the yield strength of the alloy extruded at 325 ºC was not compromised by SCC, whereas, commonly, the yield stress of Mg alloys apparently decreases in corrosion environments because SCC initiates at a lower stress. It was concluded that, in terms of ductility and strength loss, the ZX10 alloy possesses better SCC resistance compared to ZX50. This was explained by the specifics of the phase composition of these two alloys. In contrast to Mg_6_Zn_3_Ca_2_ phase particles, which dominate in the microstructure of the ZX alloys with high Zn and Ca contents, including ZX50, the Laves phase (Mg,Zn)_2_Ca, being the primary intermetallic phase in the microstructure of ZX10, is anodic with respect to the Mg matrix. It was suggested that the prioritized dissolution of this phase protects the Mg matrix from pitting-like corrosion, such as that observed in ZX50 to be initiated at cathodic particles of the Mg_6_Zn_3_Ca_2_ phase. Thus, the higher SCC resistance of the alloy ZX10 was attributed to its lower susceptibility to localized corrosion compared to ZX50. In spite of the profoundly useful information gained in the studies considered above, additional data on the SCC susceptibility of Mg-Zn-Ca alloys are still hardly demanded. For example, excellent corrosion and mechanical properties have been recently reported for the lean ZX00 alloys Mg-0.45Zn-0.45Ca (wt.%) [209] and Mg-0.6Zn-0.5Ca (wt.%) [210]. However, data on their SCC and CF resistance have not been reported as of yet. In view of the deficiency of these data, it is reasonable to perform a brief comparative survey of the SCC studies of the biodegradable Mg alloys of other systems.

**Figure 8:**
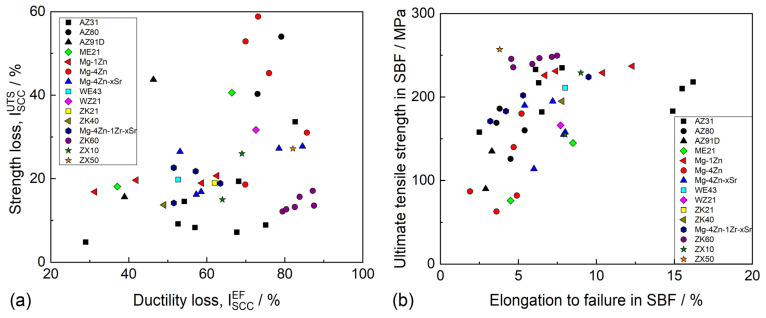
The relationships between the ductility and strength of the biodegradable Mg alloys SSRT-tested in SBFs in terms of SCC susceptibility indexes (**a**) and the absolute values of ultimate tensile strength and elongation to failure (**b**); data are imported from Table 5 and the references therein.

Table 5 summarizes the available data on the SCC of biodegradable Mg alloys SSRT-tested in different SBFs. As can be seen in this table, as well as in Figure 8a, the ductility loss index for different alloys ranges from 30 to 90%, while the strength loss scatters within the 5–60% interval. The strength loss, in most cases, lies between 10 and 30%. The alloy AZ31 demonstrates the least strength loss and also the least ductility loss when processed by ECAP [211]. It is a common practice to pay keen attention to the relative characteristics, such as the ductility or strength loss, for the assessment of the performance of a specific alloy in a corrosive environment. However, for practical purposes, it is probably more important to know the actual strength or ductility of the alloy of interest in corrosive media and not what fraction of mechanical properties has been lost due to SCC. For example, it follows from Figure 8a,b that the alloy ZX50 exhibiting quite severe strength loss still possesses superior strength in the corrosive solution among all considered alloys. Besides ZX50, the alloys ZK60, AZ31, ZX10, WE43, Mg-Zn-Zr-0.4-0.8Sr (wt.%), and Mg-1Zn have a strength above 200 MPa when SSRT-tested in a corrosion solution [163,202,207,208,211,212,213]. Among these alloys, the relatively high ductility corresponds to AZ31, ZX10, Mg-4Zn-1Zr-0.4-0.8Sr, and Mg-1Zn.

**Figure 9:**
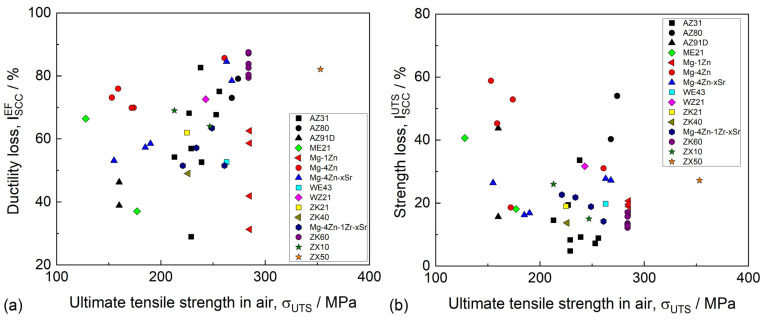
The relationships between the strength level of the biodegradable Mg alloys and their SCC susceptibility indexes by ductility (I^EF^_SCC_) (**a**) and strength (I^UTS^_SCC_) (**b**) in SBFs; data are imported from Table 5 and the references therein.

**Figure 10:**
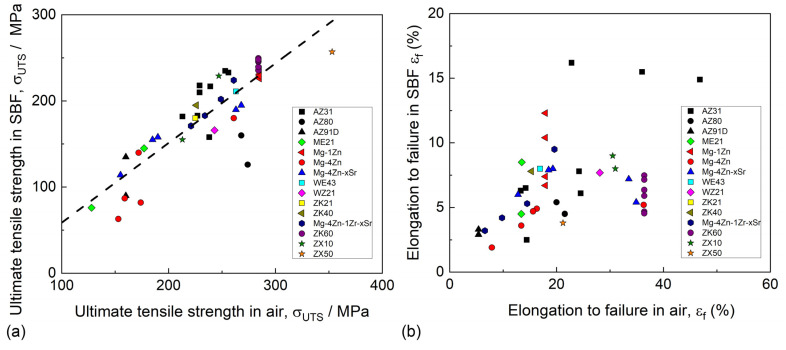
The relationships between the strength (**a**) and ductility (**b**) of the biodegradable Mg alloys in air and in SBFs; data are borrowed from Table 5 and the references therein.

It is generally accepted for most metallic materials, including steels [220,221] and nickel alloys [222], that SCC susceptibility increases with the strength level of an alloy. Mostly, this feature is relevant to the SCC associated with hydrogen embrittlement, which was also claimed to be responsible for SCC in Mg alloys. Nevertheless, as can be seen in Figure 9, neither the ductility nor the strength loss indexes of the biodegradable Mg alloys exhibit any correlation with the initial strength of the alloys. Instead, it is illustrated by Figure 10a that the strength in SBF proportionally increases with the strength in air. A similar tendency is seen in Figure 10b for ductility in SBF (though to a lesser extent than for strength), which, in general, increases along with the ductility in air. Thus, the Mg alloy with high mechanical properties in air will also likely demonstrate a high mechanical performance in SBF.

It follows from the brief survey above that the alloy AZ31 demonstrates the highest resistance to SCC in terms of both the absolute values of strength and ductility as well as the relative loss of these characteristics. However, as was mentioned in the previous subsections, the Al-containing alloys are unlikely to be used for biodegradable implants due to their unsatisfactory biocompatibility. The other factor substantially limiting the number of alloys suitable for biomedical applications is the overly high corrosion rate, which was reported for the alloys with high concentrations of Zn, such as ZK60 and ZX50 [163,207]. In contrast, the ZX10, WE43, and Mg-1Zn alloys demonstrate both high corrosion and SCC resistance [202,207,208] and can thus be considered as primary candidates to be used as Mg-based materials for biodegradable implants. One has to note that this list will likely be extended to include many other highly promising biodegradable Mg alloys such as ZX00, WZ31, and others [156,209,210]. The excellent combination of the mechanical properties of these alloys coupled with the high corrosion resistance allows us to expect a high SCC resistance, which, however, has not been reported as of yet.

### 4.2. Fatigue and Corrosion Fatigue

While the corrosion (bio-degradation) behavior of Mg alloys, particularly Mg-Zn-Ca, has been extensively studied (see Section 2) [115,149,223], the fatigue properties and, most importantly, corrosion fatigue properties have been only scarcely covered. Actually, the available data on the CF of Mg-based biodegradable alloys are even more rare than those on SCC. Furthermore, the design of virtually all implanting devices inevitably assumes geometrical irregularities and discontinuities, such as threads, holes, etc., acting as stress raisers. Moreover, these stress raisers change geometrically due to bio-corrosion processes in human body fluids, thus, in some cases, becoming more and more severe with time. Therefore, the fatigue and, particularly, the corrosion fatigue behavior of notched members made of Mg-alloys are of the utmost relevance for the implant design strategies and have to be addressed with care. Surprisingly, this has not been carried out at large yet. As a rare example, one can refer to a work by Denk et al. [224], who showed that Mg alloys (e.g., MA50) are very notch-sensitive in the high-cycle-fatigue regime, even in air. Thus, this obvious huge gap in knowledge has to urgently be filled in view of numerous clinical studies suggesting that the failure of implants is induced by fatigue (corrosion fatigue) caused by the variable load conditions experienced by implants in body liquids during their life (see, e.g., [225]). The complex interaction between the cyclic loading and corrosion of implants in the physiological environment has been the subject of many investigations. The majority of these studies have been performed on permanent implant materials [226], while biodegradable Mg-alloys have received only limited attention so far (representative references can be found, for example, in [226,227,228]). In summary, one can find a relatively small number of publications (of several tens pertinent to the corrosion fatigue properties of bio-degradable magnesium alloys), and only a few of them refer to the Mg-Zn-Ca system (see Table 6 and the citations therein). Considering that a diverse set of Mg alloys with substantially different microstructures, mechanical properties, corrosion resistances, and geometries have been tested by different authors under different testing conditions, including the solutions, frequency, loading waveforms, controlled or uncontrolled pH, steering, etc., and that preparation procedures and surface conditions (such as roughness and hardness) are often insufficiently documented, it is extremely difficult to compare the data obtained by different authors. In addition, several important methodological aspects of corrosion fatigue testing remain poorly understood and practically unexplored. Some of these aspects and their impact on test results are discussed below.

First of all, the results of corrosion fatigue tests are obviously affected by the environment, whether it be Ringer’s, Hanks’, or Kokubo’s solutions or others used in in vitro testing [229]. As has been discussed in the cited paper and above [163], the environment exerts a tremendous influence on corrosion rates. Therefore, when choosing a corrosive medium, it is necessary that its composition be as close as possible to the actual in vivo conditions. On the other hand, it would be important to standardize the corrosion fatigue practice to facilitate the exchange of the results and to make them comparable—at least the detailed composition of the test medium and the accuracy of maintaining the temperature in the test reports.

Another important aspect to consider is the design of the corrosion cell itself and its integration with the testing frame. As a matter of caution, the presence of any metallic elements in the cell can contribute to the formation of galvanic couples with the test piece, and, as shown in [230], the fatigue life and the conventional endurance limit can be significantly underestimated. Therefore, all metallic parts must be galvanically isolated, and the grips, if submerged in the cell, must be made of non-conductive materials.

An extremely important aspect of implant materials testing is the loading frequency, which, in the case of corrosion fatigue, must be regulated in a much narrower range than in conventional tests in air. For example, an increase in the frequency from 10 Hz to 80 Hz for the popular extruded alloy ZK60 leads to an increase in the endurance limit by more than 1.4 times in Ringer’s solution on the basis of 5 × 10^5^ loading cycles [231].

To illustrate the significance of the environment for the fatigue of Mg-Zn-Ca alloys, we present the results of testing the alloy Mg-1Zn-0.16Ca (ZX10). The as-cast alloy was homogenized at 450 °C for 12 h. In order to obtain a uniform fine-grained structure, the alloy billet underwent multi-axial isothermal forging (MIF) in a temperature range of 400–300 °C for five passes, with a decrease in temperature after each pass by 25 °C. The equivalent strain per pass was 1.82, and the total accumulated strain for the entire processing cycle was 9.1. Detailed information about MIF processing can be found in refs. [232,233].

Fatigue tests were carried out on an Instron ElectroPuls E1000 machine with an alternating loading cycle on corset-type specimens with a nominal cross-section of 2 × 2 mm^2^. The testing conditions were the same as those described in [231].

The typical tensile properties for the fine-grained Mg-1Zn-0.16Ca samples (mean grain size of 2.9 μm) after MIF deformation processing were: σ_0.2_ = 100 ± 5 MPa, σ_UTS_ = 205 ± 5 MPa, ε_f_ = 25 ± 2% (see Table 2). It should be noted that these results are not extraordinarily high for this alloy. Rather, the proposed thermomechanical processing should be considered as a preliminary stage in the preparation for further strain hardening, whether it be by rolling to obtain a sheet of a given thickness for the production of implants, ECAP pressing to obtain a rod of a specified diameter, rotary swaging, or other deformation processing methods. For example, in the ref. [96], for the same alloy subjected to isothermal rolling following the MIF procedure, the yield stress and ultimate tensile strength increased to 210 MPa and 260 MPa, respectively, with virtually no loss of ductility, which remained notably high (of 21% elongation to failure).

**Figure 11:**
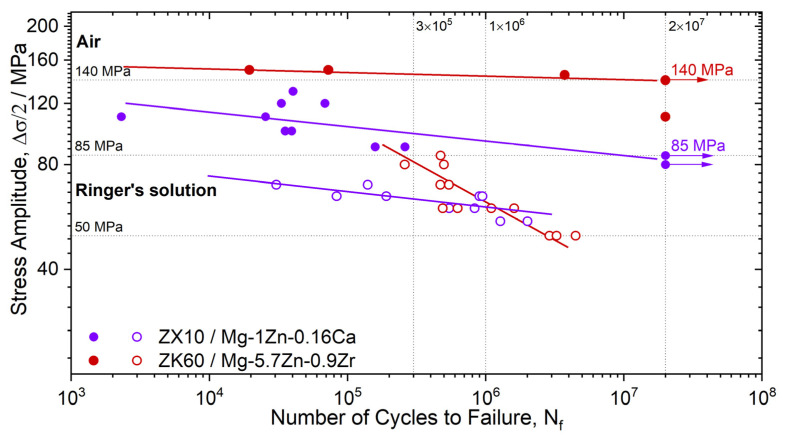
Wöhler plot for the Mg-1Zn-0.16Ca and ZK60 specimens tested at 80 Hz frequencies in air (filled circles) and Ringer’s solution (open circles). Arrows denote runouts.

The results of the stress-controlled uniaxial symmetric push–pull fatigue tests of the Mg-1Zn-0.16Ca alloy are presented in Figure 11. The results obtained for the reference conventionally hot-extruded ZX60 alloy tested under the same conditions are shown for comparison. Some other available and relevant experimental data in the literature on the corrosion fatigue behavior of high-purity Mg, Mg-Ca, Mg-Zn-Ca, and WE43 alloys are also presented in Table 6 for comparison, with the caveat that these results were obtained in different conditions. As can be seen, the endurance limit in air for the ZX10 alloy (σ_-1_ = 85 MPa) is significantly (1.65 times) lower than that for ZK60 (140 MPa) at 2 × 10^7^ cycles to failure. This is not surprising since ZK60 belongs to a class of high-strength wrought magnesium alloys, in which, due to complex alloying with Zn and Zr and subsequent processing, a very fine grain size of about 4 μm is achieved. In principle, through the deformation strengthening via SPD techniques applied for the Mg-Zn-Ca system, one can significantly improve the fatigue limit values. For example, the Mg-4Zn-0.1Ca alloy SPD-processed and fine-grained through warm ECAP followed by cold rotary swaging [20] exhibited a fatigue limit at around 120 MPa, which is a remarkable achievement for Mg alloys of this class.

**Table 6:** Tensile, fatigue, and corrosion properties of magnesium alloys.

Alloy/Composition	Processing	*d*, μm	σ_0.2_, MPa	σ_UTS_, MPa	ε_f_, (%)	σ_-1_, MPa		Ref
						Air, N_fc_ = 2 × 10^7^	Ringer, N_fc_	
ZX10/ Mg–1Zn–0.16Ca	MIF	2.9 ± 1.6	100 ± 5	205 ± 5	25 ± 2	85 ± 3	65 ± 3 (3 × 10^5^) 60 ± 3 (1 × 10^6^)	Present work
ZK60/ Mg–5.7Zn–0.9Zr	Extrusion	3.9 ± 2.2	318 ± 3	341 ± 2	14 ± 2	140 ± 3	80 ± 3 (3 × 10^5^) 60 ± 3 (1 × 10^6^)	[231]
HP–Mg	Extrusion	−	121 ± 4	208 ± 6	11 ± 2	85 (4 × 10^6^)	52 * (4 × 10^6^)	[112]
Mg–1Ca	Extrusion	−	148 ± 3	276 ± 6	14 ± 2	90 (4 × 10^6^)	70 * (4 × 10^6^)	
Mg–2Zn–0.2Ca	Extrusion	−	118 ± 6	211 ± 11	24 ± 2	83 (4 × 10^6^)	70 * (4 × 10^6^)	
WE43 alloy	Extrusion	−	217 ± 3	298 ± 4	22 ± 5	110 (10^7^)	40 * (10^6^)	[112]
Mg–1Zn–0.2Ca	Extrusion	1.2 ± 0.8	−	−	−	106 (10^7^)	60 * (5 × 10^6^)	[208]
Mg–1Zn–0.3Ca	Extrusion	7 ± 5	−	−	−	81 (10^7^)	60 * (5 × 10^6^)	
Mg–Zn–Y–Nd	Extrusion	6.9 ± 3.2	159 ± 8	229 ± 6	27 ± 1	65 (10^7^)	50 * (3.5 × 10^6^)	[234]

* Different corrosive media are used; for their complete chemical composition, the readers are referred to the literature cited.

**Figure 12:**
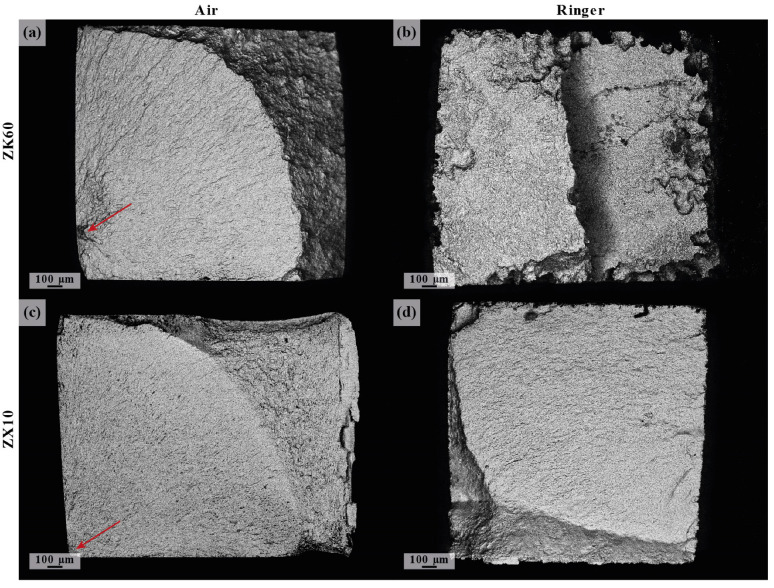
SEM images of the fracture surface after testing ZK60 (**a**,**b**) and ZX10 (**c**,**d**) in air (**a**,**c**) and in Ringer’s solution (**b**,**d**); the stress amplitudes are 140 MPa (**a**), 50 MPa (**b**), 90 MPa (**c**), and 60 MPa (**d**). The crack initiation site (**a**,**c**) is pointed to by a red arrow.

However, during cyclic loading in the biologically compatible fluid, the mechanical performance of both alloys changes drastically. For ZX10 tested in Ringer’s solution, the straight line representing the Wöhler plot in the log-log scale simply shifts towards lower stresses with the same slope as that in air. For ZK60, along with the shift towards lower fatigue lives, a sharp increase in the slope of the S-N plot is observed. Thus, within the reasonably wide range of fatigue lives up to 3 × 10^5^ loading cycles, the fatigue resistance of the high-strength ZK60 is even higher than that of ZX10. At the fatigue lives of 1 × 10^6^ cycles, the stress amplitudes are comparable for both alloys (Figure 11). The crossover point is observed around this number of cycles or at a 60 MPa applied stress amplitude. Beyond this point, in a corrosive environment, ZX60 degrades and fails much faster than ZX10. The result is counterintuitive if one can straightforwardly translate the common stress-based fatigue design principles from inert to corrosive media. Notably, the fatigue life in an inert environment is controlled by monotonic strength according to the Basquin law in the high-cycle regime: the stronger material is supposed to have a higher endurance limit (for comparable fatigue exponents). This is exactly what is seen in Figure 11 for both alloys tested in air: the stronger ZK60 (c.f., Table 6) tolerates a greater number of loading cycles to failure and exhibits a notably higher fatigue limit, while the lean MIF-processed ZX10 alloy with a lower strength at the comparable grain size fails earlier and has a lower fatigue limit. In the corrosive environment, the higher-strength alloy degrades faster, which is likely associated with the lower tolerance to corrosion-induced surface defects. It is very important to notice that the fracture surface of the specimens failed in the corrosive medium, unlike that in air (Figure 12a,c), which does not unveil any obvious signatures of fatigue failure at any applied stress amplitudes. It is practically not possible to notice any characteristic fatigue zone after testing in the corrosive solution for ZK60 (Figure 12b), and the fatigue zone in ZX10 can be recognized in ZX10 at the lowest stress amplitude (Figure 12d). The observed features are attributed to the difference in the corrosion resistance of ZK60 and ZX10 (c.f., Table 2 and refs. [96,235]; the corrosion rate in ZX60 is roughly an order of magnitude higher than that in ZX10): the lower the corrosion resistance of the material, the less clearly the fatigue features are seen on the fracture surface (c.f., Figure 12b,d).

**Figure 13:**
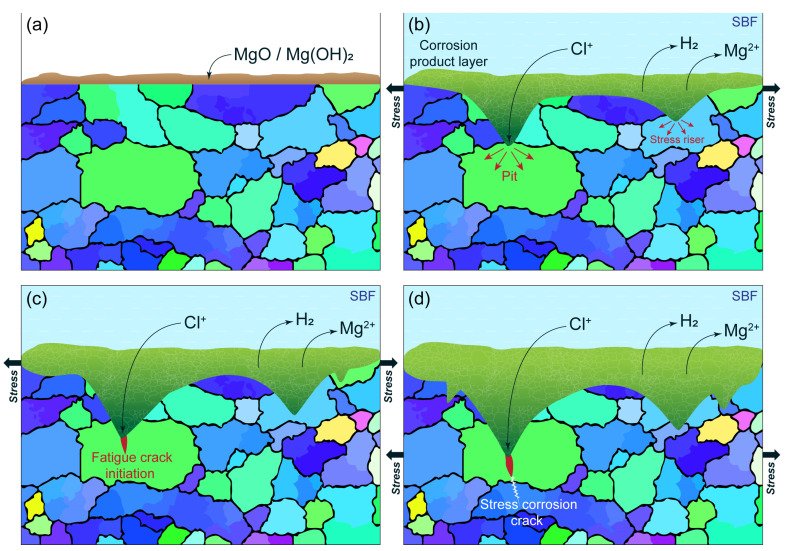
A simplified schematic illustration of the corrosion fatigue-induced damage propagation [231]. (**a**) Initial state with the MgO/Mg(OH)_2_ protective film on the surface; (**b**) advanced stage of the corrosion process under stress – the protective film is broken, resulting in the nucleation and growth of corrosion pits and the formation of corrosion product layer; (**c**) initiation of the fatigue crack at the stress concentrator created by the notch-like root of the corrosion pit; (**d**) stress corrosion cracking initiated at the sharp tip of the fatigue crack.

It was suggested that the reason for such a significant drop in the endurance of magnesium alloys in a corrosive environment is associated with the collective effect of several phenomena related to the metal–environment–load interactions resulting in less pronounced signs of fatigue failure. These phenomena co-existing throughout the test include fatigue itself, chemical corrosion, and stress corrosion cracking. The failure scenario and the corresponding sequence of failure stages are supposed to be as follows (Figure 13): (i) first, a thin oxide layer is formed on the sample surface, which can be destroyed due to cyclic loading; (ii) there is a constant flux of a new corrosive medium to the fracture sites; therefore, in places with the greatest structural inhomogeneity, corrosion pits are formed, creating a significant stress concentration at the surface; (iii) favorable conditions are met for the initiation of a fatigue crack; (iv) at the nucleated fatigue crack, the corrosion solution immediately begins to interact with the bare specimen surface, thus producing “ideal” conditions for the rapid development of SCC and fast catastrophic failure (see the preceding section). In this case, due to cyclic loading, the mechanism of SCC is activated at much lower stresses than those under static loading [173].

According to the scenario discussed above, corrosion fatigue properties are largely related to ordinary corrosion resistance: the lower the corrosion rate, the less significant the drop in fatigue strength will be. Due to the fact that alloys belonging to the Mg-Zn-Ca system are relatively corrosion-resistant, they are promising materials for temporary implants from the standpoint of corrosion fatigue.

## 5. Concluding Remarks and Outlook

We presented an overview of the substantial efforts invested into developing bio-degradable Mg-alloys, with the focus placed on the wrought Mg-Zn-Ca alloys processed by different thermo-mechanical routes devised to improve their properties profile, notably by taking the benefits of severe plastic deformation techniques for microstructure refinement. Without the intention to review the wealth of results on the mechanisms underlying the mechanical or corrosion behavior of bio-degradable alloys, we provided a concise summary of existing properties, outlined existing gaps in the knowledge, and looked into the challenges faced by practitioners dealing with Mg alloys targeted for biomedical implant applications. The purpose was to equip the reader with a review of the subject and to offer insights into the further developments needed to promote the Mg alloys towards their successful application in clinical practice. We illuminated the complexity of the effect of processing on the tensile and fatigue properties. Furthermore, we show that many specific scientific and technological challenges in this area reflect the fundamental problems in understanding the strength and plasticity of magnesium alloys.

Great expectations have been placed on achieving superior mechanical properties of magnesium alloys with the aid of severe plastic deformation techniques. We demonstrated that an exceptional balance of high strength, ductility, and fatigue resistance is achievable in Mg-Zn-Ca alloys after thermo-mechanical processing involving either a conventional extrusion or a variety of SPD techniques. Furthermore, the impressive mechanical performance of these alloys is paired with a good bio-corrosion resistance, which can be well controlled in a wide range by careful alloy cleaning from trace impurities and tailoring the fine uniform microstructure. Issues faced in attempts to assess the corrosion rate reliably are highlighted, and guidelines are provided on how to mitigate them.

In our view, the acute needs are still high in further understanding the mechano-chemical interactions of biodegradable alloys; specifically, a fundamental understanding of the extremely hazardous stress corrosion cracking and corrosion fatigue mechanisms in biological fluids has yet to be achieved for the guided alloy design against these damaging processes, which inevitably occur in deployed temporary implants. It is fair to say that the mechanisms for these phenomena are not fully understood, which calls for more research on the subject.

It would be naïve, of course, to believe that this brief review comprehensively covers the wide, multifaceted area of research that has been carried out on bio-degradable alloys in general and on Mg-Zn-Ca in particular. Exciting areas which remain intact in the present overview are the surface treatment, the formation of nano-scaled and gradient microstructures in the sub-surface layer of magnesium alloys, and the formation of a surface relief for the enhancement of corrosion resistance, cell attachment, and proliferation. Of particular interest are the interaction of magnesium alloys with living matter and the significance of the surface topology and the microstructure in this interaction. We intentionally did not touch on an important section of extensive in vivo studies associated with the biological implications of Mg structures implanted into living tissues, their biological interactions with living cells, and their mechanical integrity in human body fluids. This research is being conducted on such a massive scale worldwide that the need for a dedicated review of this topic is greater than ever. We hope that, even with this limitation, the reader now has a coherent image of a fascinating new research arena and that the present overview will stimulate further interest in developing innovative biocompatible magnesium alloys and the routes of their industrial-scale processing, enabling enhanced bio-medical and mechano-chemical performance.

## Figures and Tables

**Table 1:** Typical Properties of Implant Materials.

Material	Density g/cm^3^	Young’s Modulus/GPa	0.2% Yield Strength /MPa	Ultimate Tensile Strength /MPa	Specific Strength /MPa·cm^3^/g	Tensile Elongation at Break (%)	Fatigue Strength *** /MPa	Reference
Cortical bone	1.6–2.0	15–31	31–71	72–151	-	-	-	[9]
αFe	7.87	211	150	240	44.5	49	130	[10]
316LVM (Medical Grade)	8.0	188	405	515	64	68	400	[11,12,13]
CP Ti (Grade 2)	4.5	103	275	345	76	20	300	[12,13,14]
CP Ti (Grade 4)	4.5	105	485	550	122	15	425	[12,13,14]
Ti-6Al-4V ELI (α + β)	4.43	114	770	840	190	15	427	[12,13,14,15]
Ti-12Mo-6Zr-2Fe (β)	5.0	74	895	930	186	15	730	[16]
CoCr (Wirobond^®^ C) *	8.5	180	440	780	92	16	-	manufacturer
CoCr (L605) **	9.27	242	424	1021	110	12	343	manufacturer
Mg	1.74	44	21	90	52	2–6	28	[17]
Mg-1Zn-2Y (at.%) (LPSO)	1.84	45	610	-	332	5	-	[18]
Mg–8Gd–3Y–0.4Zr (wt.%)	<2	43	650	710	355	4.5	-	[19]
Mg-4Zn-0.15Ca (wt.%)	<2	45	348	381	190	5	120	[20,21]

* Co—bal., Cr 24.8, W 5.3, Mo 5.1, Si < 1, Fe < 1, Ce < 1, C < 0.02 wt.% (BEGO Bremer Goldschlägerei Wilh. Herbst GmbH & Co., Bremen, Germany); ** Co—bal., Cr 20, W 15, Ni 10, Si 0.4, Fe 3, Mn 1.5 C < 0.1 wt.%; *** Fatigue strength refers to 1 × 10^7^ symmetrical loading cycles of unnotched samples in ambient air at room temperature; LPSO denotes the alloy with the long periodic stacking ordered microstructure.

**Table 3:** Corrosion rate of biodegradable Mg-based alloys in Hanks’ solution at 37 °C.

Composition	Alloy	Manufacturing Features	Grain Size (μm)	Measuring Method	pH-Level/Adjustment Method	Duration (for Immersion Tests)	Corrosion Rate (mm/year)	Ref.
Mg-Al-Zn	AZ91	As-cast	-	Hydrogen evolution	7.0/bubbling CO_2_	283 h	6.8	[141]
						168 h	3.8 ± 1.5	
				Weight loss		168 h	6.2 ± 2	
		-	-	Weight loss	7.3/solution replacing (every 8 h)	168 h	0.61 ± 0.017	[142]
				PDP		-	0.072 ± 0.005	
	AZ31	-	-	Weight loss		168 h	0.84 ± 0.025	
				PDP		-	0.077 ± 0.005	
Mg-Y	1Y	Extrusion	3.4	Weight loss	≤8.9/without adjustment	72 h	0.25 ± 0.05	[143]
					≤10.15/without correction	360 h	0.3 ± 0.1	
	3Y		10		≤9.65/without correction	72 h	0.85 ± 0.1	
					≤10.5/without correction	360 h	1.95 ± 0.25	
Mg-Y-Zn	WZ21	Extrusion	-	Weight loss	7.0/bubbling CO_2_	336 h	2.7	[141]
						168 h	0.46 ± 0.11	
			-	Hydrogen evolution			0.23 ± 0.22	
	WZ31	As-cast	150	Hydrogen evolution	7.2–7.8/solution replacing (auto)	168 h	1.86	[144]
				Weight loss			8.65	
		Multiaxial isothermal forging + pressing	3	Hydrogen evolution			2.10	
				Weight loss			2.47	
Mg-Zn-Ga	4Zn4Ga	Equal channel angular pressing	90/10	Hydrogen evolution	7.3–6.3/without correction	192 h	0.16 ± 0.06	[119]
				PDP	-	-	1.55 ± 0.1	
	4Zn4Ga 0.2Ca		10	Hydrogen evolution	7.3–6.3/without adjustment	192 h	0.37 ± 0.07	
				PDP	-	-	2.48 ± 0.05	
	4Zn4Ga 0.3Y	Equal channel angular pressing	10	Hydrogen evolution	7.3–6.3/without adjustment	192 h	0.22 ± 0.07	
				PDP	-	-	1.75 ± 0.05	
	4Zn4Ga 0.3Nd	Equal channel angular pressing	10	Hydrogen evolution	7.3–6.3/without adjustment	192 h	0.30 ± 0.07	
				PDP	-	-	1.72 ± 0.05	
Mg-Zn-Ca (include Mg-Zn and Mg-Ca)	ZX10	As-cast	400	Hydrogen evolution	7.2–7.8/solution replacing (auto)	168 h	4.08 ± 2.3	[144]
				Weight loss			5.56 ± 2.5	
		Multiaxial isothermal forging + pressing	4	Hydrogen evolution			2.5 ± 0.32	
				Weight loss			3.7 ± 0.86	
	0Zn1Ca	As-cast	~200	Weight loss	7.2–7.4/HCl + NaOH	720 h	3.16 ± 0.5	[145]
	1Zn1Ca		~300				2.13 ± 0.2	
	2Zn1Ca		~100				2.38 ± 0.3	
	3Zn1Ca		~100				2.92 ± 0.5	
	4Zn1Ca		~100				4.42 ± 1	
	5Zn1Ca		~100				6.15 ± 1.5	
	6Zn1Ca		~100				9.21 ± 1.5	
	2Zn0Ca	As-cast	388	Weight loss	7.4/H_3_PO_4_	168 h	0.3	[146]
	2Zn0.2Ca		~250				0.37	
	2Zn0.4Ca		~200				0.6	
	2Zn0.8Ca		175				1.76	
	2Zn0.7Ca	Extrusion (220 °C)	~3.6	Hydrogen evolution	≤9/without adjustment	192 h	0.55 ± 0.3	[147]
				PDP	-	-	2.3 ± 0.3	
		Extrusion (300 °C)	~6.6	Hydrogen evolution	≤9/without adjustment	192 h	0.3 ± 0.25	
				PDP	-	-	2.55 ± 0.05	
	4Zn0.7Ca	Extrusion (220 °C)	~4.3	Hydrogen evolution	≤9/without adjustment	192 h	0.96 ± 0.12	
				PDP	-	-	1.92 ± 0.4	
		Extrusion (300 °C)	~7.2	Hydrogen evolution	≤9/without adjustment	192 h	1.2 ± 0.3	
				PDP	-	-	1.8 ± 0.8	
	2Zn	Extrusion	24.8	Hydrogen evolution	7.4/without adjustment	168 h	0.064	[148]
				PDP	-	-	0.1	
	2Zn0.1Ca		6.5	Hydrogen evolution	7.4/without adjustment	168 h	0.095	
				PDP	-	-	0.11	
	2Zn0.3Ca		1.1	Hydrogen evolution	7.4/without adjustment	168 h	0.172	
				PDP	-		0.39	
	3Zn0.4Ca	As-cast	-	Hydrogen evolution	7.4/bubbling CO_2_	0–130 h	1.04	[149]
						130–336 h	3.88	
	4Zn0.5Ca	Squeeze cast	86.8	Weight loss	-	72 h	1.2 ± 0.4	[150]
						144 h	2.3 ± 0.1	
						216 h	2.9 ± 0.3	
				PDP	-	-	0.326 ± 0.143	
	4Zn1Ca	Homogenization treatment	197	PDP	-	-	6.73	[151]

**Table 4:** Corrosion rate of biodegradable Mg-Zn-Ca alloys in other corrosion media with 37 °C.

Alloy	Corrosion Media	Manufacturing Features	Grain Size (μm)	Remarks	Measuring Method	pH-Level/Adjustment Method	Duration (for Immersion Tests)	Corrosion Rate (mm/year)	Ref.
Mg-1.25Zn-0.8Ca	Kokubo solution	As-cast	-	-	Weight loss	≤9.8/without adjustment	144 h	1.62	[152]
					PDP	-	-	4.12	
Mg-2.5Zn-0.8Ca			-	-	Weight loss	≤9.3/without adjustment	144 h	2.9	
					PDP	-	-	5.07	
Mg-4Zn-0.8Ca			-	-	Weight loss	≤10/without adjustment	144 h	4.13	
					PDP	-	-	7.26	
Mg-3Zn-0.2Ca	0.9% wt. NaCl	As-cast (quenching in water)	-	-	Hydrogen evolution	-	48 h	3.06 ± 0.14	[153]
					Weight loss			4.86 ± 0.25	
					PDP		-	0.34 ± 0.01	
Mg-3Zn-0.5Ca			-	-	Hydrogen evolution	-	48 h	3.2 ± 0.23	
					Weight loss			5.11 ± 0.33	
					PDP		-	0.38 ± 0.04	
Mg-3Zn-0.8Ca			-	-	Hydrogen evolution	-	48 h	4.84 ± 0.21	
					Weight loss			7.43 ± 0.65	
					PDP		-	0.51 ± 0.07	
ZX11	FBS	As-cast	54.1		Weight loss	≤8/daily replacing	24 h	0.22 ± 0.01	[154]
							72h	0.22 ± 0.07	
							336 h	0.29 ± 0.09	
		Rotary swaging	4.5/4.8	-			24 h	0.28 ± 0.01	
							72h	0.22 ± 0.01	
							336 h	0.44 ± 0.09	
Mg-1Zn-0.2Ca	Ringer’s solution	As-cast	185	-	Hydrogen evolution	7.4/H_3_PO_4_	168 h	8.5 ± 1.3	[96]
					Weight loss			7.6 ± 0.7	
		Multiaxial isothermal forging	2.9		Hydrogen evolution			1.1 ± 0.24	
					Weight loss			1.8 ± 0.5	
		Multiaxial isothermal forging + rolling	2.2		Hydrogen evolution			1.3 ± 0.26	
					Weight loss			1.7 ± 0.3	
		Extrusion	36/5.2	Bimodal Structure (Large grains/Small grains)	Hydrogen evolution			3.2 ± 0.5	
					Weight loss			1.6 ± 0.4	
ZX10	0.9% wt. NaCl	Multiaxial isothermal forging + pressing	4	-	Hydrogen evolution	7.6 max/without adjustment	168 h	1.75 ± 0.1	[155]
					Weight loss			3 ± 0.25	
ZX10	Ringer’s solution	Extrusion	2.1		Hydrogen evolution	7.4 ± 0.4/H_3_PO_4_	168 h	3.2	[156]

**Table 5:** The SCC performance of the biodegradable Mg alloys in SBFs.

Alloy	Manufacturing Features	Grain Size, d/µm	Ultimate Tensile Strength, σ_UTS_/MPa		Elongation to Failure, ε_f_, %		I^EF^_SCC_, %	I^UTS^_SCC_, %	Strain Rate, s^−1^	Corrosion Solution	Ref.
			Air	SBF	Air	SBF					
WZ21	Extrusion	7	243	166	28.1	7.7	73	32	3 × 10^−7^	m-SBF	[207]
ZX50		4	353	257	21.2	3.8	82	27			
WE43		15	263	211	16.9	8	53	20			
AZ31	Dry machining	24	256	233	24.5	6.1	75	9	3.5 × 10^−6^	SBF	[213]
	Cryo machining	24	253	235	24.2	7.8	68	7			
Mg-4Zn	High-strain rate rolling	4	261	180	36.3	5.2	86	31	6.7 × 10^−7^	Hanks	[214]
Mg-4Zn-0.1Sr		3.6	263	190	34.9	5.4	85	28			
Mg-4Zn-0.2Sr		3.3	268	195	33.5	7.2	79	27			
AZ31	As-received	27.5	213	182	14.2	6.5	54	15	3.5 × 10^−6^	SBF	[211]
	Equal channel angular pressing, one pass	8.3	229	218	22.8	16.2	29	5			
	Equal channel angular pressing, two passes	6.8	229	210	36	15.5	57	8			
	Equal channel angular pressing, four passes	6.5	227	183	46.8	14.9	68	19			
Mg-1Zn	Wrought	17	285	226	17.9	6.7	63	21	1 × 10^−6^	PBS	[202]
				231		7.4	59	19		m-SBF	
				229		10.4	42	20		DMEM	
				237		12.3	31	17		BCS	
Mg-4Zn	As-cast	80	172	140	15.6	4.7	70	19	6.7 × 10^−7^	Hanks	[215]
Mg-4Zn-0.1Sr		45	190	158	19.3	8	59	17			
Mg-4Zn-0.2Sr		57	185	155	18.5	7.9	57	16			
Mg-4Zn-0.4Sr		62	155	114	12.8	6	53	26			
Mg-4Zn	As-cast	482	153	63	13.4	3.6	73	59	3.6 × 10^−6^	SBF	[216]
	T4	398	174	82	16.3	4.9	70	53			
	T6	420	159	87	7.9	1.9	76	45			
ME21	As-cast	325	128	76	13.4	4.5	66	41	1 × 10^−6^	Hanks	[203]
	Equal channel angular pressing	6	177.1	145	13.5	8.5	37	18			
ZK40	Bi-directional forging	8.2	226	195	15.3	7.8	49	14	1 × 10^−6^	m-SBF	[212]
ZK40-0.4Sr		6.7	261	224	19.6	9.5	52	14			
ZK40-0.8Sr		5.7	249	202	14.5	5.3	63	19			
ZK40-1.2Sr		3.7	234	183	9.8	4.2	57	22			
ZK40-1.6Sr		3.7	221	171	6.6	3.2	52	23			
ZK21	Extrusion	3	-	-	-	-	62	19	3.1 × 10^−7^	m-SBF	[217]
ZX10	Extrusion 325 C	1.2	247	229	30.5	9	64	15	3.1 × 10^−7^	m-SBF	[208]
	Extrusion 400 C	7	213	155	31	8	69	26			
AZ91D	As-cast	-	160	135	5.4	3.3	39	16	3.1 × 10^−7^	Hanks	[218]
			160	90	5.4	2.9	46	44		Hanks + BSA	
AZ80	Hot-rolling	50	268	160	20	5.4	73	40	1 × 10^−6^	SBF	[219]
			194	176	13.6	4.7	65	9			
			274	126	21.54	4.5	79	54	5.3 × 10^−7^		
			180	129	11	4.6	58	28			
ZK60	Extrusion	3	284	239.5	36.4	5.9	84	16	5 × 10^−6^	0.9%NaCl	[163]
			284	249.5	36.4	7.485	79	12	5 × 10^−6^	Ringer	
			284	235.5	36.4	4.675	87	17	5 × 10^−6^	Hanks	

## Data Availability

The experimental data obtained by the authors are available from the corresponding author upon reasonable request.
